# Modern Approaches in Wounds Management

**DOI:** 10.3390/polym15173648

**Published:** 2023-09-04

**Authors:** Simona-Maria Tatarusanu, Florentina-Geanina Lupascu, Bianca-Stefania Profire, Andrei Szilagyi, Ioannis Gardikiotis, Andreea-Teodora Iacob, Iulian Caluian, Lorena Herciu, Tudor-Catalin Giscă, Mihaela-Cristina Baican, Florina Crivoi, Lenuta Profire

**Affiliations:** 1 Department of Pharmaceutical Chemistry, Faculty of Pharmacy, University of Medicine and Pharmacy “Grigore T. Popa” of Iasi, 16 Universitatii Street, 700115 Iasi, Romania; tatarusanu.simona-maria@email.umfiasi.ro (S.-M.T.); florentina-geanina.lupascu@umfiasi.ro (F.-G.L.); andreea.panzariu@umfiasi.ro (A.-T.I.); caluian.iulian@d.umfiasi.ro (I.C.); lorena-antoanina_herciu@d.umfiasi.ro (L.H.); 2 Research & Development Department, Antibiotice Company, 1 Valea Lupului Street, 707410 Iasi, Romania; 3 Department of Internal Medicine, Faculty of Medicine, University of Medicine and Pharmacy “Grigore T. Popa” of Iasi, 16 University Street, 700115 Iasi, Romania; bianca-stefania.profire@d.umfiasi.ro; 4 Advanced Research and Development Center for Experimental Medicine (CEMEX), University of Medicine and Pharmacy “Grigore T. Popa” of Iasi, 16 University Street, 700115 Iasi, Romania; andrei.szilagyi@umfiasi.ro (A.S.); ioannis.gardikiotis@umfiasi.ro (I.G.); 5 Department of Obstetrics and Gynecology, Faculty of Medicine, University of Medicine and Pharmacy “Grigore T. Popa” of Iasi, 16 University Street 700115 Iasi, Romania; gisca_tudor-catalin@d.umfiasi.ro; 6 Department of Pharmaceutical Physics, Faculty of Pharmacy, University of Medicine and Pharmacy “Grigore T. Popa” of Iasi, 16 University Street, 700115 Iasi, Romania; mihaela.baican@umfiasi.ro

**Keywords:** wound healing, wounds assessment tools, biopolymers, smart hydrogels, stimuli-responsive

## Abstract

Wound management represents a well-known continuous challenge and concern of the global healthcare systems worldwide. The challenge is on the one hand related to the accurate diagnosis, and on the other hand to establishing an effective treatment plan and choosing appropriate wound care products in order to maximize the healing outcome and minimize the financial cost. The market of wound dressings is a dynamic field which grows and evolves continuously as a result of extensive research on developing versatile formulations with innovative properties. Hydrogels are one of the most attractive wound care products which, in many aspects, are considered ideal for wound treatment and are widely exploited for extension of their advantages in healing process. Smart hydrogels (SHs) offer the opportunities of the modulation physico-chemical properties of hydrogels in response to external stimuli (light, pressure, pH variations, magnetic/electric field, etc.) in order to achieve innovative behavior of their three-dimensional matrix (gel–sol transitions, self-healing and self-adapting abilities, controlled release of drugs). The SHs response to different triggers depends on their composition, cross-linking method, and manufacturing process approach. Both native or functionalized natural and synthetic polymers may be used to develop stimuli-responsive matrices, while the mandatory characteristics of hydrogels (biocompatibility, water permeability, bioadhesion) are preserved. In this review, we briefly present the physiopathology and healing mechanisms of chronic wounds, as well as current therapeutic approaches. The rational of using traditional hydrogels and SHs in wound healing, as well as the current research directions for developing SHs with innovative features, are addressed and discussed along with their limitations and perspectives in industrial-scale manufacturing.

## 1. Introduction

Wounds are injuries which occur when sudden, rash, and mostly unexpected accidents affect the integrity of skin. The injury may result from different causes such as cuts, crushes, thermal/radiation burns, or surgical events, and could be extended from superficial damages, which affect only the skin’s layers, to deep tissues (muscles, nerves, and blood vessels) destruction [1]. The main concern in wounds management is the high risk for chronicity. Among the patients hospitalized for acute conditions, 25–50% present or develop wounds during hospitalization, with high risk of infection and chronicity [2]. The rate of infection of surgical incisions is 3–4% and causes an 5% additional increase in mortality [3,4].

It is estimated that 2% of the population in developed countries suffer from a chronic wound during their lifetime [5]. In Europe, according to the European Wound Management Association (EWMA), the prevalence of chronic wounds is 3–4/1000 (1.5–2.0 million out of 491 million inhabitants), the incidence being 4 million patients/year [6,7]. Patients suffering from chronic wounds may experience continuous pain, loss of mobility and functionality, increased stress level, social isolation, depression, anxiety, prolonged hospitalizations, impairment of work capacity, and negative financial impact. Moreover, if chronic wounds are located in the lower limbs (e.g., diabetic foot ulcer), amputation occurs in 85% of patients [8] and the mortality rate increases to 40% after 5 years [9].

Currently, a wide range of pharmaceutical products such as creams, gels, ointments, powders, pastes, and patches, with healing, antimicrobial, and/or moisturizing effects are available for wound management. The medical approach of wound care must be correlated with patients’ individual reactivity and preferences in order to maximize the treatment compliance.

## 2. Pathophysiology of Wound Healing

**Figure 1:**
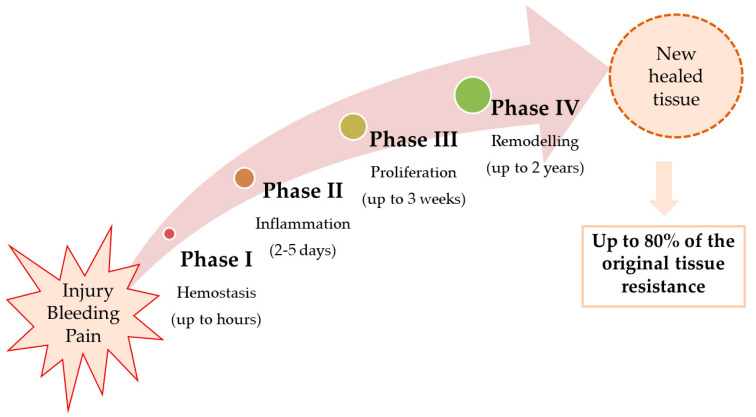
The phases of the wound healing process.

Immediately after injury, a cascade of physiological reactions is triggered in order to restore the physical and functional integrity of the affected area [10]. Wound healing involves four steps: hemostasis, inflammation, proliferation, and remodeling (Figure 1).

As a result of the inflammatory and hemostasis phases, release of cytokines, growth factors, and migration of leukocytes (in the first 2–5 days), phagocytosis and debridement (late inflammation), and vasoconstriction, platelet aggregation, and coagulation are occur [11].

The proliferative phase lasts between 2 days and 3 weeks and includes the following: *granulation* (fibroblasts fill the defect with collagen fibers; new blood vessels are formed); *contraction* (collagen fibers are reorganized, the edges of the wound come closer to each other, reducing the size of the defect), and *epithelialization* (epithelial cells proliferate, covering the defect). If adequate tissue oxygenation is not achieved, the fibroblast function and angiogenesis are disrupted and the healing process is impaired [11].

The remodeling phase continues after the proliferative phase for up to 2 years, when new, thicker collagen fibers, oriented in the direction of the force lines, are formed. The extracellular matrix is in a remodeling process and the activity of metalloproteases is increased. Epithelial cells and fibroblasts enter apoptosis and the scar that is formed at the end of this phase assures up to 80% of the strength of the original tissue [12].

The ability of the body to restore its homeostasis after a traumatic event depends on maintaining the balance between the endogenous substances and the cells that mediate the healing process. Any inconsistency between the physiological parameters can cause the disruption, prolongation, or complication of the healing phases through infection, chronicity, and expansion of the injured tissue [5,10]. The healing process is influenced by several factors such as mass index body, local anatomy, associated medication, infection, dysmetabolism, and patient compliance. In addition, the associated comorbidities (diabetes mellitus, high blood pressure, obesity, autoimmune diseases, peripheral vascular dysfunctions) interfere with healing physiological process and can impact the outcome of the treatment [13].

The common characteristic of chronic wounds is a persistent inflammatory phase, which is responsible for reducing phagocytic capacity and bactericidal effect, as well as increasing cellular and microbial residues at the site of aggression, which delays the re-epithelialization process [14]. Thus, if the healing of acute wounds is completed within 3 weeks [2], for chronic wounds the healing ranges between 1–3 months, affecting the normal recovery of the affected area [2,3].

**Figure 2:**
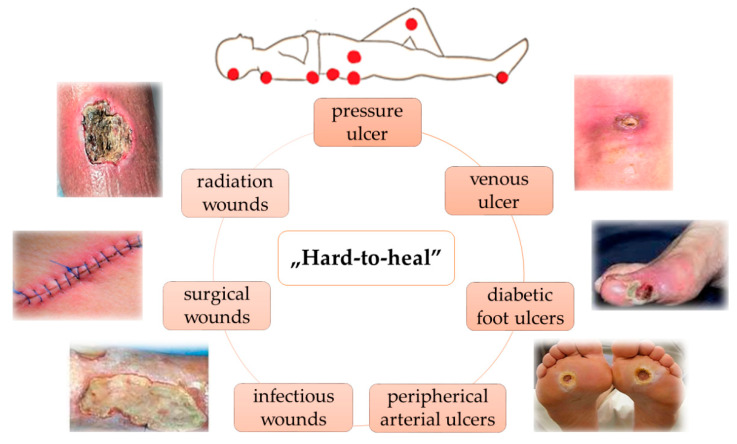
The most common chronic wounds, according to WHF classification.

The Wound Healing Foundation (WHF) classifies chronic wounds in several categories, depending on their etiology: pressure ulcers, venous ulcers, diabetic ulcers, and arterial insufficiency ulcers as well as radiation, surgical, and infectious wounds (Figure 2).

## 3. Wounds Care Management Protocol

Given the diversity of causes, types of wounds, and the individual characteristics of patients, wound management is difficult to standardize. However, it is imperative that this process be carried out as objectively as possible, so that the established treatment protocol can be effective. The treatment should be closely related to type of wound and its healing phase and in correlation with the patient’s comorbidity. Thus, a detailed anamnesis about the time of the wound appearance, previous treatments and therapies, the evolution until presentation in the medical unit, the associated diseases and their treatments, and other relevant information for the patient’s status (e.g., nutritional screening) should be noted. It is also important to know that an impaired wound may favor the appearance of systemic pathologies, which trigger a vicious circle for the healing process [4]

The evaluation of a wound is primarily based on visual observation which emphasizes the location, size, and depth of the wound, drainage, and the type of tissue in the injured area. Secondly, the wounds must be evaluated for microbial loading. The commonly known signs of infection such as erythema, edema, pain, and fever may not always be present [15].

**Figure 3:**
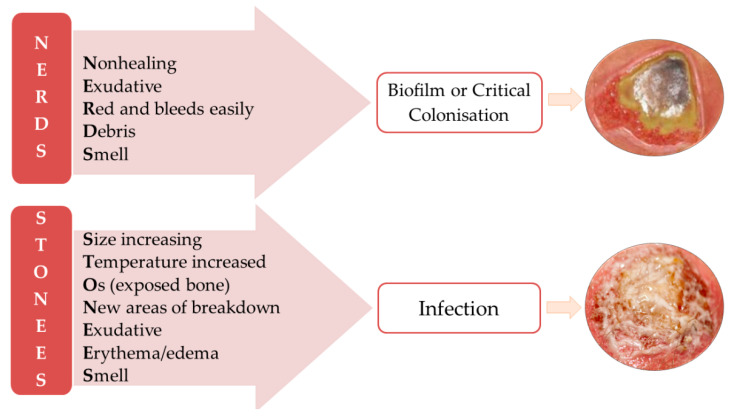
Mnemonics for clinical assessment and differential diagnostic between infected and biofilm-colonized chronic wounds.

Two mnemonics are recommended for infected chronic wound identification NERDS, with 73% sensitivity and 80.5% specificity when three criteria are met, and STONEES, which has 90% sensitivity and 69.4% specificity when at least three signs are observed (Figure 3). When NERDS criteria are met, biofilm is present or the wound is critically colonized. If the STONEES are observed, then the chronic wound is infected [16,17].

The quantitative determination of the microbial load of a chronic wound is recommended to be performed by biopsy and a positive result means more than 100,000 colony forming units. Sometimes, even with the best care, some acute or chronic wounds do not respondto the treatment properly. In these cases, no sign of healing is seen even after six weeks of treatment and the physicians call them atypical wounds. These wounds could have a variety of etiologies, such as metabolic, genetic, malignant, infectious, and inflammatory [17].

**Figure 4:**
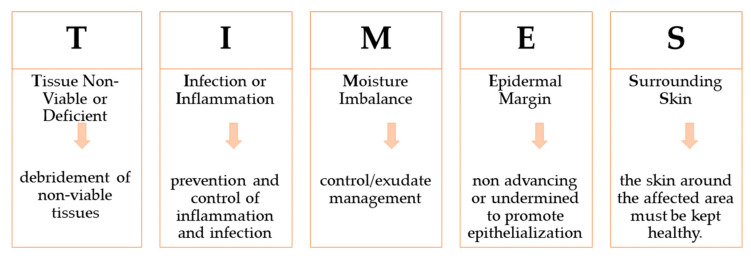
TIMES concept for evaluation of critical parameters during wound healing phases.

To support the medical healthcare professionals, the **TIMES** acronym was implemented two decades ago, and it is still used (Figure 4). It standardizes the indicators used in wound evaluation and gives information to establish a treatment plan [18].

The first step in wound care is debridement, which consists in the removal of non-viable and dead cells and/or tissue. Studies showed that after perseverant debridement, the time healing of chronic wounds is halved [18]. In a cohort study of 154,664 patients with more than 300,000 wounds, for which the debridement was made at least once a week, an overall healing rate of 70.8% was reported [19].

The removing of biofilm is also important, even more than debridement, because it causes the blocking of the healing process in the inflammatory phase [20]. According to the National Institute of Health (NIH), biofilms are responsible for 80% of infections in chronic wounds [12]. Biofilms are composed of numerous multicellular colonies which produce an extracellular matrix to hold them together.

Moreover, the reestablishment of the moisture balance is mandatory. When the optimal moisture is achieved, the risk of microbial colonization is lower and the healing process is accelerated, in both acute and chronic wounds [11,12]. For a draining or a dry wound, the optimal moistness is reach by applying a proper dressing. This prevents the desiccation of a wound’s bed andholds the appropriate moisture in order to accelerate the proliferation and remodeling phases [21,22].

The appropriate wound treatment is established in accordance with the principles of the TIMES protocol. Thus, the debridement, medical or surgical control of persistent inflammation, application of dressings (including negative pressure), protection of surrounding skin with dressing, and appropriate emollients will be decided upon according to the TIMES principles [12,23,24].

The Triangle of Wound Assessment recommends to extent the TIMES concept beyond wounds’ edges and outlines the importance of inspecting three zones in the affected area: wound edge, wound bed, and periwound area. For the wound bed and edge, the evaluation is similar to the TIMES guideline. The examination of the periwound area gives information on skin status around the affected area such as presence of maceration, excoriation, dry skin, hyperkeratosis, callus, and/or eczema [25,26]. Keeping the skin around the wound in a healthy state is crucial for reducing the extension of the affected area and to shorten the healing time.

The best practice statement of Effective Exudate Management (EEM) was published in 2013 to help healthcare professionals in the assessment of exudate color, odor, and viscosity, in order to select the proper dressing. The guideline was focused on the understanding of the exudate role in the healing process and how its characteristic should vary based on inflammation, capillary breakage, microbial colonization, and protein content [27].

The Pressure Ulcer Scale of Healing (PUSH) is often used to assess the evolution of pressure ulcers over time in terms of dimensions of affected area, exudate amount, and tissue type. This tool has a high accuracy; it is very fast and reliable, being validated by the National Pressure Ulcer Advisory Panel [28].

## 4. Wound Care Treatment Approaches

A proper wound care treatment should correlate a systemic approach with topical treatment and adequate nutritional support to assure the success of healing. Systemic treatment mainly targets comorbidity or septicemia risk, while topical treatment focuses on modulation of molecular reactions which occur in pathophysiological pathways on the damaged area. The final scope of topical approach is the restoration of skin integrity with minimum scar damages (as fibrosis, hypertrophies, keloids, red fibrous lesions, contractures) [19].

Wound dressings have evolved over time, from clay tablets (2500BC) and cotton gauze (manufactured for the first time in 1891) to modern dressings, whose development began with proving (in 1948) the beneficial effect of a moist chamber for venous ulcers. Nowadays, dressings include more than 2000 pharmaceuticals products, from topical creams, gels, and ointments, to sophisticated medical devices such as bacteria-killing lasers and 3D steam cells printers [19,29]. Approximately 30% of the wound care products market is held by wounds cleansers, followed by the dressings segment [30].

An optimal wound dressing requires a balance between its benefits, safety for the patient, and cost-effectiveness. The ideal wound dressing should be biocompatible, biodegradable, and have the optimal permeability for water vapor to ensure and maintain a favorable environment during the entire healing process [31]. In addition, it should fit perfectly to the wound’s shape, but not be adherent to the wound surface, ensure protection from microbial contamination, mechanical, and thermal stress, and should optimize moisture in the injured area [32]. The topical treatment for chronic wounds raises substantial costs for the healthcare system. The global annual average financial burden is estimated to rise to almost USD 100 billion per year. For example, in UK, the average cost per year is GBP 5.3 billion, which is comparable with the average 5 billion GBP/year for obesity managing, according to the National Institute for Health and Care Excellence (NICE) [24,33]. In addition, in the USA, where there are approximately 6.5 million patients diagnosed with chronic wounds, the cost on the health system is approximately 25 billion USD/year [6,7].

Wound dressings are divided intotraditional or conventionaldressings (such as cotton gauze, bandages, lint, plasters) and modern multifunctional dressings (such as foams, films, hydrocolloids, hydrogels, nanocomposites) [10,32,34]. Conventional dressings are known as passive wound dressings and are useful to cover and stabilize modern dressings. The latter dressings are defined as interactive products which modulate the entire healing cascade by controlling the damaged tissue microenvironment (moisture, temperature, pH), stimulating the granulation and reepithelization processes, and inhibiting microbial growth [35,36].

### 4.1. Hydrogels as Modern Wound Dressings

Hydrogels were documented for the first time in 1960, when Wicherke and Lim prepared a gel based on hydroxymethylacrylate for medical application [37]. Nowadays, hydrogels still fascinate biomedical researchers, having applications in tissue engineering (bone, cartilage, and muscles), cell growth, wound healing, controlled drug release, biosensors, and medical devices [34,38,39]. Hydrogels are defined as three-dimensional (3D) hydrophilic polymer networks capable of encapsulating large amounts of water or biological fluids. As a result of their hydrophilic character, oxygen permeability, ability of diffusion, cell and molecules adhesion, and ease of use, hydrogels demonstrate important beneficial effects for wound care [34,40].

In many aspects, hydrogels are considered ideal wound dressings because they mimic the skin structure, promoting the growth factor synthesis and autolysis process. Moreover, their ability to incorporate and release a wide variety of active pharmaceutical ingredients (APIs) makes hydrogels suitable for the management of draining painful wounds, radiation wounds, minor burns, or dry wounds [37].

**Figure 5:**
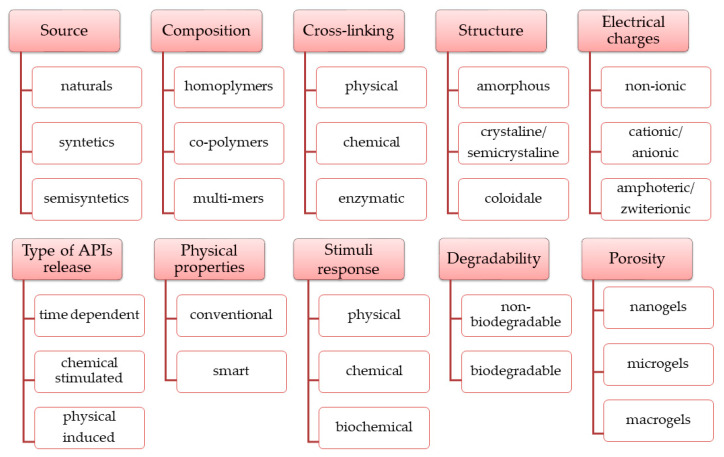
Hydrogels classification, based on polymers characteristics and technological manufacturing process criteria.

Over time, extensive research has led to numerous types of hydrogels with varied physico-chemical properties according to several criteria. The main categories of hydrogels (Figure 5) were established according to the raw materials sources and polymeric composition, physico-chemical properties, release conditions of the APIs embedded in the 3D matrix, and other properties [34,37,41,42].

Hydrogels have several advantages, such as the following:3D structure, similar to the extracellular matrix, which plays a key role in cell proliferation, debridement, and exchange of vital substances for epidermal cells; hydrophilicity, associated with hydrophilic groups of the polymer matrix: NH_2_, -COOH, -OH, -CONH_2_, -CONH-, and -SO_3_H; water absorption capacity, due the porosity degree; high degree of flexibility similar to human tissue, due the water content; biodegradability and biocompatibility [43,44]. Along with the positive aspects, some drawbacks reduce the applicability of hydrogels in medical practice. These include poor mechanical stability, high risk for microbial contamination (except chitosan-based hydrogels), toxicity potential, degradation, and variable release profiles of embedded APIs [44,45].

The mechanical properties of hydrogels are closely related to several factors, such as the cross-linking method, polymer type, ratio of polymer: cross-linking agent, structure of the embedded APIs, and storage conditions [46]. An indicator of mechanical stability of hydrogel is viscosity, which decreases over time, because its 3D structure is affected through degradation reactions (oxidation, reduction, hydrolysis, free radical interactions) [47]. Hydrogels based on covalent interactions are more stable compared to hydrogels based on hydrogen bonds or physical interactions [48]. Innovative hydrogels, based on dynamic covalent bonds (dynamic Schiff bases), combine excellent stability over time with a versatile stress behavior [49]. The dynamic covalent bonds break when shear forces (injection, application) are applied or in presence of stressful stimuli (variations in pH, temperature, electric/magnetic field), without breaking of the hydrogel’s backbone. When the stress conditions are removed, the hydrogels partially or totally recover their 3D structure and mechanical performance [49,50,51].

The high water content of hydrogels provides a proper environment for microbial contamination (e.g., *Pseudomonas aeruginosa*, *Staphylococcus aureus*, *Escherichia coli*, *Candida albicans*), which can delay the healing process [46]. Moreover, microbial contamination causes changes in the aspects of hydrogels (color, smell) and negatively influences their mechanical stability [43]. In order to reduce the risk of contamination, different materials with antimicrobial effects (polymers, cross-linking agents, APIs, preservatives) or different cross-linking methods were used. For example, 3D matrices obtained through Schiff bases dynamic bonds have proven antifungal and antibacterial properties [50,52].

The aldehydes and epoxide compounds, used as cross-linking agents, lead to hydrogels with excellent mechanical stability, but the by-products as well as the unreacted compoundsshow high toxicity, such as genotoxicity, cytotoxicity, or carcinogenicity [53]. Moreover, these compounds may affect the healing process by extending the inflammatory phase [53,54,55].

In order to improve the kinetics release of APIs embedded in the 3D polymer matrix, different types of carriers (nanoparticles, nanospheres, nanofibers, liposomes, microparticles) are combined with novel cross-linking methods [56,57].

### 4.2. Smart Hydrogels as Innovative Wound Dressings

Extensive researchhas expanded the functional roles of hydrogels to smart materials that not only protect the wounds but have the ability to influence all phases of the healing process. The concept of smart was mentioned for the first time in 1949 by Kunh et al., in their research on polyacrylic acid 3D networks, capable of mimicking muscle contractions under acidic conditions and absorbing huge quantities of water when alkaline substances were added [58].

**Figure 6:**
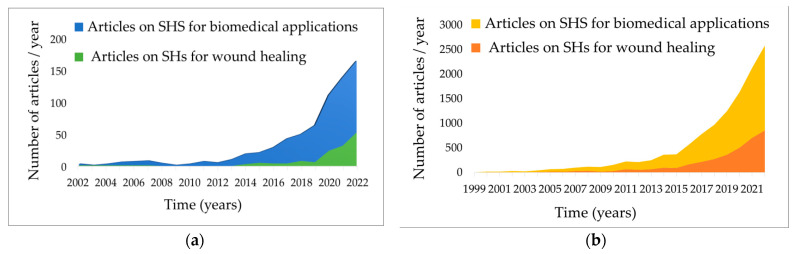
The articles relating to SHs from PubMed (**a**) and Science Direct (**b**) databases.

According to PubMed and Science Direct databases, in the last two decades, the amount of research on smart materials for biomedical fields has increased year by year, summing up to 8500 reviews and original articles in 2022, including more than 3600 on smart hydrogels (SHs) dedicated to wound care management (Figure 6).

In wounds approach, SHs bring some important benefits such as shortenedhealing time and improved complication prevention, which lead to better results and increased quality of life for the patients [38,40,59].

Both natural and semi-synthetic/synthetic polymers may be successfully used for SHs formulation, using physical or chemical gelation methods [36].

Among the most widely used chemical cross-linking methods are Schiff-base reactions, Michael addition reactions, free-radical polymerization of end-functionalized macromers, high-energy radiation, and enzymatic reactions [55]. Chemical cross-linking results in highly stable structures, with process controllable features such as time gelation, degree of cross-linking, and pore size and pore density of the matrices [41]. The main drawback of these methods is toxicity risk, due the by-products resulting from cross-linking reactions [53]. In addition, the cross-linkers may interact with bioactive components (cells, pharmaceutical active proteins—enzymes, hormones, growth factors) embedded in the hydrogel matrix and affect their therapeutic target [55].

Physical cross-linking involves weak interactions such as hydrogen bonds, van der Waals forces, ionic and hydrophobic interactions, crystallizations, chain entanglements and coordination bonds, protein interactions, and freezing–thawing and heating/cooling cycles [41]. The main limitation of these methods results from gelation reversibility under a wide range of conditions which may be modulate in favor of targeted applications of the final product, or, on the contrary, may induce stability problem over time [55].

Moreover, 3D and 4D bioprinting technics (ink writing, digital light synthesis/ processing, stereolithography, fused deposition modeling) were tested for the manufacturing of SHs with excellent mechanical properties (deformability, flexibility). The low productivity due to slow speed of printing, restrictions due the biopolymers stability to heating, and expensive equipment are drawbacks that needs to be addressed before implementing these methods on a microscale and industrial scale production of SHs via 3D/4D printing [60].

The target of multifunctional SHs in wound care is to overcome the drawbacks of traditional hydrogels and to improve their therapeutic performance using the ability to reversibly modify their 3D structure in response to one or more external stimuli [61]. The comparative characteristics of traditional hydrogels versus SHs relevant for manufacturing and performance of wound care products are summarized in the Table 1

### 4.3. Stimuli-Responsive Hydrogels

**Figure 7:**
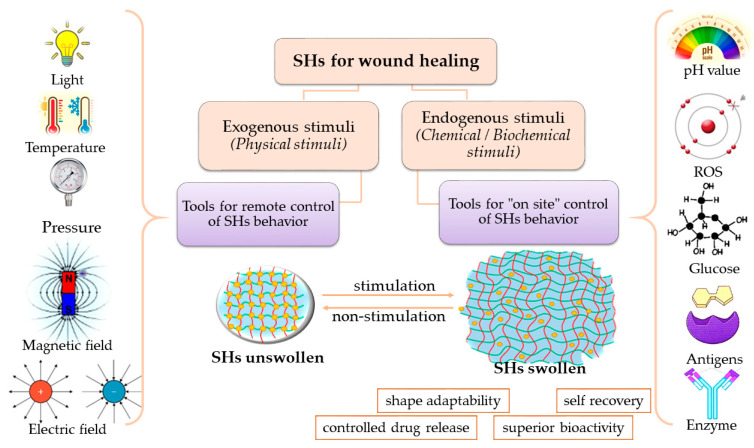
Stimuli that enhance the performance of SHs on the wound healing process.

Depending on the nature of the gelling agents and the manufacturing method, the hydrogels respond to different physical (temperature, light, pressure, ultrasounds, exposure to magnetic and electric fields) or chemical/biochemical stimuli (pH variations, chemicals like reactive oxygen species, glucides, enzymes, antigens, ionic charge, solvents). Depending on their sources, the stimuli are also divided into endogenous or exogenous stimuli [39]. Endogenous stimuli result from physiological reactions or from pathological pathways: (i) presence of glucose, enzymes, and ions in the human body, (ii) pH variations in blood and exudates as result of infections, (iii) generation of reactive oxygen species in damages tissues. Exogenous stimuli (temperature, light, magnetic/electric field, ultrasounds, pressure) are used as remote tools for non-invasive temporal and spatial control on hydrogels behavior (Figure 7) [36].

Based on stimuli action, several changes in physical state and/or chemical properties such as gel–soil transition, sequential deformations, shape adapting, viscosity variations, conductivity, hydrophilicity, and degradation are produced [69]. These changes are translated into beneficial effects in medical applications such as modulation of drug release kinetics, in situ gelation, enhancing biocompatibility and over-time stability, and over-stress recovery ability. Once the stimuli are removed, the 3D networks may return to their original state, acting as smart materials with applications in tissue engineering and regenerative medicine, biosensors, drug delivery/stem cells systems, contact lenses, and implants [59,62]. In addition, SHs are able to adapt to the wound shape for a perfect fit on the affected area, which means that the damaged tissue has an intimate contact with healing care products [70,71].

The SHs may respond to one or more stimuli, which endows the product with gradually modified behavior correlated to spatial-temporal changes in each phase of wound healing.

#### 4.3.1. Physical Stimuli-Responsive Hydrogels

##### Temperature-Responsive Hydrogels

Hydrogels based on natural/semi-synthetic/synthetic polymers (CS, methylcellulose, gelan gum, poloxamers, polyethylenglycol (PEG), poly(N-isopropylacrylamide) (PNIPA), poly(vinyl alcohol) (PVA), poly(N-vinylcaprolactam) and their derivatives) respond to temperature variations with sol–gel transitions due to thermal disturbance of the pre-existing equilibrium between the hydrophilic and hydrophobic units in the 3D networks [35,39]. These hydrogels, which mimic the extracellular matrix, can be easily functionalized with bioactive molecules, and may exhibit a controlled degradation rate [36].

In the wound recovery process, temperature plays a critical role in the reactions occurring in the affected area. A temperature around 37.8 °C is desired for optimal healing and deviations of ±2.2 °C indicate an ischemic or inflammatory process, which leads to tissue deterioration. The temperature-responsive hydrogels must undergo sol–gel transition at physiological temperature in order to be suitable for wound care.

A catechol modified quaternized CS, poly-lactic acid (PLA), and PEG were used as gelling agents to develop temperature responsive injectable hydrogels. The modified CS endowed the hydrogel with antibacterial properties and wet-tissue-adhesiveness. In addition, the functionalized CS lowered the gelation point to 32 °C, near the physiological value. The in vivo study showed that the thermosensitive hydrogel properly seals the wound area and promotesaccelerated healing [72]. Because thermo-responsive hydrogels behavior only lies on the amphiphilic properties of the polymers’ backbones, these formulations are considered non-toxic, cost-effective, and easy obtained. However, in absence of a cross-linker agent, the stability overtime may be limited.

A thermosensitive wound dressing was obtained from combination of CS with Pluronic P123 (a triblock symmetric poloxamer) and gelatin, which was loaded with curcumin for its antioxidant and anti-inflammatory effects. At temperatures higher than 30 °C, it undergoes a sol–gel transition, depending on gelatin concentrations, and gradually releases the curcumin on the wound site. An in vivo wound model revealed that the hydrogel stimulates fibroblasts and immune cells migration and neoangiogenesis in the damaged tissues [73].

A combination of poloxamers with different molecular weights was used to develop a thermosensitive hydrogel for controlled release of *Nocardia rubra* cell wall skeleton. The formulation has proved excellent beneficial effects on in vitro and in vivo studies for diabetic wound healing, as stimulation of vascular endothelium, expression of structural proteins, activation of phosphatidylinositol, and protein kinase [74].

PNIPA and pullulan were associated to develop a 3D matrix in which silver NPs were loaded. The release profile of silver, and thus the antimicrobial activity, were depended on the wound temperature, the sustained released being maintain for 48h. In addition, in vitro tests demonstrated nontoxicity and excellent swelling properties, the hydrogels being suitable for wounds care [75].

A thermo-responsive multifunctional DNA-based hydrogel was design through cross-linking of DNA units with polyethyleneimine and black phosphorus quantum dots, and procyanidin B2 were embedded in its matrix. The finished product showed excellent uptake capacity for exudative wounds, proper adhesiveness, self-healing capacity, and antibacterial and antioxidant activity. The pro-inflammatory M1 macrophages were proliferated into repairing M2 phenotype and myeloid cells were attracted to the damaged areas. In consequence, angiogenesis was sustained and skin nerves, follicles, and hair regeneration were promoted [76].

##### Pressure-Responsive Hydrogels

Pressure relief is one of the main concerns of the treatment strategy in diabetic foot ulcers and pressure ulcers pathology. The pressure occurs by frictions during physical activities and environmental pressure and causes tissues necrosis and delayed healing [61]. Using pressure-sensitive dressings, secondary injures are prevented in wound care, with benefits regardinghospitalization time and the healing period.

Sodium carboxymethyl cellulose, alginate, CS, and PNIPA were successfully used in the development of hydrogels that are able modify their viscosity and swelling capacity under pressure variations [38,77]. For natural polysaccharides-based hydrogels, the reactivity to pressure was related to electrostatic repulsions that occur between the chemical groups with the same charge (e.g., NH_3_^+^ in CS) which control the swelling rates and the impact the osmotic pressure inside the 3D network [77]. A liquid hydrogel based on PVA and acrylamide with antibacterial and anti-inflammatory properties was designed to monitor movements and pressure status in real-time for bed-ridden patients. The hydrogel possesses very high pressure sensitivity from 9.19 kPa^−1^, which allows to easy evaluate the pressure of the injured site and avoid exceeding the limits and secondary tissues destructions. In addition, the hydrogel shows excellent mechanical strength, enhances cell proliferation, and promotes tissue remodeling and regeneration [78].

##### Light-Responsive Hydrogels

An aseptic environment is crucial for wounds healing and it is a huge challenge to ensure non-contamination of the affected tissue. The exposure to different types of light (ultraviolet or visible light) was demonstrated to improve the wound healing of chronic wounds [79]. The high intensity of visible light has an efficient bactericidal effect and, followed by exposure of the wound to low intensity illumination, promotes optimal wound closure [80].

Alginate, CS, hydroxypropyl methyl cellulose, carbomers, and methacrylates were used to design near-infrared, ultra-violet, and visible-light-responsive hydrogels. Their formulation involves grafting photoactive groups (azobenzene, nitrobenzyl derivatives, diaryne, spiropyrans, photoreversible dimerization groups such as coumarin, anthracene, and pyrimidine derivatives) on polymers’ backbones in order to absorb photons with different wave length energy and trigger phase transitions or chemical reactions. The absorbed light energy usual must be converted into thermal energy for the changes in hydrogels 3D networks to occur [35,38]. Light-responsive hydrogels proved to be excellent options for design matrices, with controlled release of APIs and exposure to light triggers enhancing the antimicrobial and antioxidant activity [81].

Dodecyl moieties were added to CS and tungsten disulfide nanosheets were included as a photothermal agent to develop a photo-sensitive hydrogel, which could release ciprofloxacin on wound sites when near-infrared red light is applied. The hydrogel proved efficient antimicrobial activity and antioxidant effects which prevent prolonged pro-inflammatory state in damaged tissues [82]. Poly(2-hydroxyethyl methacrylate) covalently cross-linked with polyurethane was used to develop an antimicrobial light-sensitive hydrogel. The nitric oxide (NO) photoactive donors (nitrosyls), hydrogen peroxide and methylene blue, were incorporated in hydrogels as antimicrobial agents. The data suggest the superior outcome of using the light-sensitive hydrogel over hydrogen peroxide solutions for cleaning the chronic wounds [83].

Light-sensitive polyurethane nanofibers dressing for wounds beds and bandages with tetraphenylporphyrin as photosensitizer were developed. Use of this dressing in a study in which 162 subjects with chronic diabetic foot ulcers were enrolled showed inhibition of *Staphylococcus aureus*, *Pseudomonas aeruginosa*, and *Escherichia coli* growth in the wound site proportional with visible light exposure. Furthermore, an average of 35% shrinking of wounds size and 71% wound-related pain reduction was observed on tested groups compared to the control group [84].

A nanodelivery light-responsive system with sinoporphyrin sodium (a hematoporphyrin-like photosensitizer) and fibroblast growth factor embedded into poly(lactic-co-glycolic acid) nanospheres was developed based on a carboxymethyl CS and sodium alginate hydrogel matrix. This scaffold showed promising antibacterial activity and antibiofilm effects against multidrug-resistant *Staphylococcus aureus* on severe burn wound models, which are photoirradiation-conditions-depended [85].

However, the prolonged ultra-violet light exposure of injured tissue during a potential treatment with this type of SHs may raise some concerns regarding patients’ safety, due to the carcinogenicity of UV rays. In addition, some injectable light-sensitive SHs could be designed for deep wounds and the clinical applicability of these formulations is limited by lack of penetration of UV in deep tissues.

##### Magnetic-Responsive Hydrogels

The application of a magnetic field is a promising approach in wounds treatment strategies, being a non-invasive alternative and without significant side-effects [86]. The mechanism of healing under external applied magnetic field is still unclear. There are studies which showed a 25% faster healing of induced wounds in animal [87].

Alginates, xanthan gum, hemicellulose, and PNIPA were used to design magnetic-sensitive hydrogels with iron oxide (Fe_3_O_4_, Fe_2_O_3_), ferrites (MnFe_2_O_4,_ CoFe_2_O_4_), or alloys (FePt), included in different 3D matrices as drug delivery sensing or tissue engineering and wound healing applications [38]. The main advantage of these hydrogels is the smart and precise remote response (deformation, motion, or heat generation) under magnetic exposure [86]. Controlling the magnetic pulse applied on/off, the hydrogels could enhance or inhibit drug release and protein absorption in the wound site [88].

The development of SHs gives the ability to modify the APIs behavior, as their hydrophilicity and permeability is an ongoing concern for topical formulations [59]. For example, a hydrogel based on alginate derivate and MXenes (two-dimensional inorganic compounds that consist of atomically thin layers of transition metalcarbides, nitrides, or carbonitrides)has demonstrated controllable release of antibioticsin response to photo and magnetic stimuli. This hydrogel showed the successful healing of full-thickness cutaneous and subcutaneous infected wound in a rat model [89].

##### Electric-Responsive Hydrogels

Alginate, agarose, HA, xanthan gum, PEG, polydimethylsiloxane, and poly(lactic-co- glycolic acid) were used to develop biocompatible electroconductive hydrogels for controlled drug delivery, wound dressing, biosensors, and tissue regeneration, which are able to modify their swelling behavior and structure flexibility upon application of an electric stimuli [35]. The electric charge could be transported by either an electrolyte in hydrogel composition, by polymers with intrinsic conductivity (gelatine, CS, polyaniline, polypyrrole), or by conductive particles (carbon, metals) embedded in a hydrogels network [90].

As is known, cell migration starts in the first days when injury occurred, and it is essential for a normal healing process. The electrical field simulates and coordinatescell migration, cell differentiation, and regeneration in chronic and non-healing wounds [91]. The mechanisms are not yet clearly understood, but the studies outlined the activation of some intercellular polyamines and cell membrane channels [92]. Using covalent or non-covalent cross-linking methods, conductive responsive hydrogels were prepared. The in vivo tests sustain the benefits of electric-responsive hydrogels in all steps of wound healing, showing a hemostatic effect, proliferation of fibroblasts, granulation stimulation, angiogenesis, and increased collagen synthesis [90].

A hydrogel based on quaternized CS-graft-polyaniline and benzaldehyde-functionalized PEGdemonstrated self-healing ability, good hemostatic properties, excellent adhesiveness, and more than a 99% bactericidal effect on *Staphylococcus aureus* and *Escherichia coli* [93]. HA functionalized with dopamine and reduced graphene oxide, as a photothermal agent, was used to prepare a conductive hydrogel that mimics the human skin and proved in vivo enhancement of antimicrobial activity of doxycycline under near-infrared radiation [94].

#### 4.3.2. Chemical Stimuli-Responsive Hydrogels

##### pH-Responsive Hydrogels

The pH of wound exudates reaches values in the alkaline range (7.0–9.0) if it becomes infected, while the pH of healthy skin is in the 4.5–6.5 range. Moreover, the values of pH higher than 6.5 favor the growth of *Staphylococcus* spp., *Pseudomonas aeruginosa*, *Klebsiella* spp., and formation of biofilms, which are commonly met in impaired wound healing.

The polymers that contain acidic (carboxyl, hydroxyl) or alkaline (amino) groups (as poly(vinyl alcohol)–PVA, alginate, hyaluronic acid–HA, chitosan–CS, albumin, pectin, gelatin, polymethacrylic acid, polyacrylic acid, and phenylboronic acids) are suitable for development of pH-sensitive hydrogels, due the protons exchange between oppositely charged groups. Polymers could also be functionalized by inclusion of amino acidmoieties on the backbones to achieve pH responsiveness hydrogels [35]. For example, an alginate-based hydrogel in acidic pH has non-ionized carboxyl groups in the network and exhibits shrinkage with insignificant swelling rate. If the pH is towards alkaline (e.g., in chronic wounds), the carboxyl groups become ionized and, due the electrostatic repulsion, the hydrogel starts to uptake important amounts of fluids [95]. These types of hydrogels are most suitable for exudative wounds management such as diabetic wounds and pressure ulcers. In addition, due the swelling/deswelling behavior in response to pH variations, these formulations offer good opportunities for modulation of the drug delivery and 3D cell culture.

Wang et al., developed a pH-sensitive hydrogel to monitor the wound status using colorimetry. Based on a three-step monitoring model and using a machine learning technology, the monitoring algorithm has 94% accuracy. Moreover, the hydrogel has shape adaptability for a perfect match to the wound surface [96]. Another study reports the development of pH-sensitive hydrogels, based on carboxylated agarose and tannic acid, and zinc ions as cross-linker, which exhibited antibacterial and anti-inflammatory effects. The release of tannic acid was pH-controlled, being negligible at pH > 7 and sustained at acidic values [97].

An in situgelling injectable hydrogel, sensitive to temperature and pH, was developed based on PEG and poly (sulfamethazine ester urethane). The multiblock copolymer is a solution at room temperature with an alkaline pH and is transformed to a gel inhuman body conditions (37 °C and pH of 7.4). DNA-bearing polyplexes were included in the copolymer solution and the controlled released from the 3D matrix formed in situ was assessed. Moreover, the hydrogel showed excellent adhesive properties and was easy to remove from the affected skin, being suitable for wound healing treatment [72].

A dual-stimuli-responsive (pH and oxidative stress) phenylboronic acid-modified HA dynamically cross-linked hydrogel, loaded with tannic acid and silver NPs as APIs, was developed. The hydrogel proved cytocompatibility, self-healing, antibacterial, anti-oxidative, and injectable abilities, which means it is very promising for wound care applications [98]. Electron-rich furan groups were grafted on HA backbones in order to obtain a redox-responsive hydrogel based on maleimide bonds, for controlled release of APIs. Polyethylene glycol, which form disulfide bonds between HA backbones, was used as redox-responsive cross-linker.

CS grafted with carboxymethyl moiety and oxidized HA were used to develop self-healing and pH-responsive hydrogels through dynamic Schiff base interactions. Taurine was loaded into the hydrogel matrix for its anti-inflammatory effect and proved to enhancehealing by stimulating cell migration and reducing the inflammatory phase on an in vivo diabetic wound model [99].

A dual-responsive 3D network with high drug loading capacity was developed by cross-linking of alginate with glutamic acid cysteine dendrimer and polyethylene glycol. The doxorubicine hydrochloride, which was embedded in the hydrogel matrix, was released by over 76%, depending on gluthatione and pH variations [100]. Efficient antibacterial activity on *Staphylococcus aures* and *Escherichia coli* was proved by a pH-responsive physically cross-linked citric acid/alginate-based hydrogel. Vancomycin loaded in a hydrogel matrix was best released in acidic conditions, up to 86%, on zero-order kinetics [101].

A multi-stimuli-responsive PVA-based hydrogel, physically cross-linked, loaded with gentamycin and cyanine dyes (cyanine 3 and cyanine 5) as silica NPs, was developed. This hydrogel was able to detect the microbial colonization in wound beds through delivery of cyanine due to pH-responsive fluorescence resonance energy transfer, and to release the gentamycin from cleavable linkers in response to near infrared light [102]. PVA and poly 6-acrylamidohexanoic acid were dually physically cross-linked through hydrogen bonds and crystallizations in a pH-dependent self-healing 3D matrix. The hydrogel exhibits high mechanical stability, undergoes structural breakage in acidic conditions, and recovers its entire arrangement in neutral condition. In addition, it uptakes different amounts of fluids in pH variations due to the breakage and restoration of hydrogen bonds, which allows the control of moisture level on the wound healing process [103].

A double-networked multi-stimuli-responsive PNIPA and keratin-based hydrogel was designed through covalent crosslinking and ionic interactions. The hydrogel matrix was loaded with chlorhexidine, the release of which was regulated by the hydrogel’s response to pH and temperature variations and the presence of reactive species of oxygen in the wound site. Moreover, the antimicrobial effect of the hydrogel loaded with chlorhexidine was superior to chlorhexidine alone, due its sustained release. In vivo evaluation of wound healing proved to be superior to commercial Tegaderm (based on polyurethane), due to neoangiogenesis and superior collagen synthesis [104,105].

##### Reactive-Oxygen-Species-Responsive Hydrogels

Reactive oxygen species (ROS) which act as messengers in cell migration (immunocytes and non-lymphoid cells) are involved in angiogenesis of new blood vessels on the injured area and stimulate the pathogens phagocytose. The benefit key of ROS in wound healing is maintaining equilibrium between the production and the scavenging of free radicals. In chronic and non-healing wounds, an over-expression of ROS is observed, and it contributes to prolonged inflammation [106].

ROS-responsive hydrogels could be obtained using polymers functionalized with thioethers, which due the presence of sulphur in molecules could undergo phase transitions when exposed to ROS. Thioethers are converted into sulfoxides in mild oxidative conditions or into sulfones under strong conditions, which results in modification of polymers solubility and disruption of the hydrogel network arrangement. Selenium-containing polymers act similarlyto thioethers, with even higher sensitivity to ROS, and could be oxidized to selenoxides and selenones, resulting in changing the polymer behavior from hydrophobic to hydrophilic [107].

Gallic acid was used as antioxidant in a gelatin-hydroxyphenyl propionic acid hydrogel, which significantly reduced the destruction of dermal fibroblast in H_2_O_2_-induced oxidative stress. L-arginine microparticles were incorporated in an ROS-responsive hydrogel to promote wound healing. Under ROS stimulation, a sustained quantity of NO is generated from L-arginine, which promotes the fibroblast and macrophage chemotaxis, collagen synthesis, and skin tissue regeneration [108].

Alginate functionalized with phenylboronic acid was used as a backbone for an injectable dual (pH and ROS)-stimuli-responsive hydrogel with self-healing ability, in which amikacin as an antibacterial, and naproxen as an anti-inflammatory drug, were embedded via preloaded micelles. The in vitro experiments showed that the hydrogel effectively killed bacteria by amikacin release, with an inhibition rate up to 90% for *Staphyloccocus aureus* and 98% for *Pseudomonas aeruginosa*. Furthermore, naproxen was also controllably released from the ROS-responsive micelles under pH 5.0 and H_2_O_2_ in vitro [109]. Carboxybetaine/sulfobetaine dextran were used to obtain antioxidant self-healing zwitterionic hydrogels, with the ability to scavenge free hydroxyl radicals and to inhibit *Staphylococcus aureus* and *Escherichia coli* [110]. Fluorescein isothiocyanate conjugated bovine serum albumin as API was encapsulated within HA-based hydrogel, yielded through the Diels–Alder reaction with a maleimide group containing redox-responsive PEG-based cross-linker. When 1,4-dithiothreitol was added, the disulfide bonds between HA backbones were broken, and the API loaded into the 3D matrix was rapidly released [111].

##### Glucose-Responsive Hydrogels

High levels of glucose, matrix metalloproteinases, and pro-inflammatory mediators are involved in impaired healing in diabetic chronic wounds. Hyperglycemia prolongs the healing processes by promoting microbial colonization, sustaining biofilms formation, and impairing tissue neovascularization. Glucose-responsive hydrogels have gained much interest in the controlled release of bioactive compounds as antioxidants and anti-inflammatory molecules, for topical wounds treatments [112].

PEG functionalized with phenylboronic acid (PBA) derivatives or lectins (concanavalin A) are promising polymers for the development of SHs that respond to elevated local glucose concentration. PBA interacts with diols in polymer backbones and results in 3D matrices. When glucose levels increase, the cross-linked network is disrupted because glucose binds to PBA at pH higher than PBA acid pKa (around 9.0). Grafting amino groups on polymers functionalized with PBA enable the scaffolds to function at physiologic pH by reducing the pKa of boronic acid derivatives, translated in modifying the swelling behavior and undergoing gel–sol phase transitions [113].

PVA and CS functionalized with PBA were used to develop a temperature-sensitive 3D matrix loaded with insulin and celecoxib, capable of quickly adapting to irregular wounds shape at 37 °C. The release of APIs is regulated by high concentrations of glucose and matrix metalloproteinase-9 (MMP-9) in exudate, resulting in their local down-regulation and inflammation decrease, cell migration, and proliferation in diabetic full-thickness-wound models. In addition, the hydrogel possesses special rheological behavior, combining a fluid-like mobility at 37 °C for the intimal cover of irregular-depth injuries with a solid-like elasticity at 25 °C, which ensures wound protection against environmental stress [114]. HA was also functionalized with PBA as a glucose-sensitive molecule and mixed with PEG diacrylates to obtain a hybrid network in which myricetin was loaded as an antioxidant active compound. Interactions between phenol groups of myricetin and functionalized HA result in dynamic bonds which endow the hydrogel with the ability to release myricetin depending on the local glucose level. In addition, the ROS-scavenging effect was higher than 80% and superoxide dismutase and glutathione levels were increased, while expression of pro-inflammatory mediators (IL-6) was reduced. Moreover, angiogenesis and tissue remodeling were accelerated, which make this hydrogel promising for diabetic wound care [113]. In a hyperglycemic wound environment, the glucose-oxidase turns glucose into glucuronic acid and hydrogen peroxidase, lowering the local pH.

A self-healing injectable hydrogel based on co-assembling of zinc ions, organic ligands, and deferoxamine mesylate was loaded with glucose-oxidase. The release of zinc ions and deferoxamine from the hydrogel improves cell proliferation, angiogenesis, reduces oxidative stress, and suppresses prolonged inflammation. In vivo evaluation on diabetic wound models showed optimal re-epithelization with collagen deposition and inhibition of microbial growth [115].

Glucose-sensitive hydrogels have shown excellent benefits for diabetic chronic wounds, but their clinical applicability is questionable because of lack of predictability of the triggered in vivo response when different molecules and/or microorganism are involved. In addition, the stage of healing in chronic wounds and patient comorbidities may also affect the responsiveness of hydrogels.

### 4.4. Polymers for SHs Formulation

#### 4.4.1. Biopolymers

##### Polymers from Natural Sources

**Figure 8:**
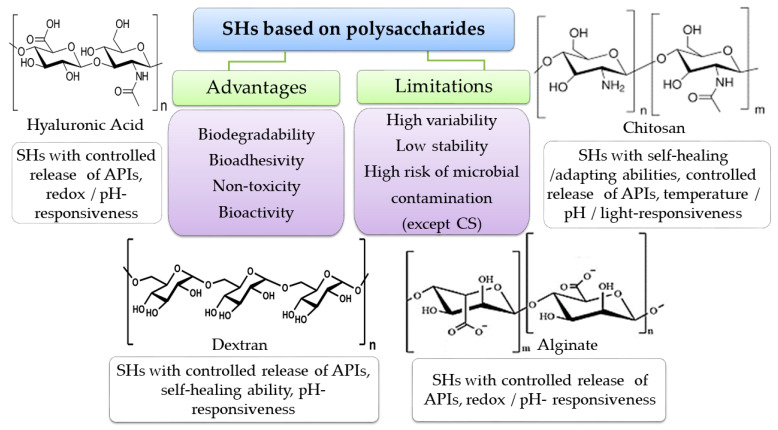
Natural polymers used on SHs formulation for wound managementapplications.

Natural polymers, such as CS, HA, alginates, dextran, cellulose, agarose, gelatin, collagen, cyclodextrin, fibrin, pullulan, polylysine, pectin, carrageenan, and chondroitin sulfate, may be obtained from a wide variety of natural sources and could successfully design versatile SHs. Natural-polymers-based hydrogels have superior biodegradability and biocompatibility compared to those based on synthetic polymers. In addition, natural polymersendowhydrogels with inheritance bioactivities such antimicrobial and antioxidant effects, similarities with endogenous molecules, and cytocompatility [116]. Moreover, the complex structure of natural polymers offers plenty of chemical and physical structural modulation opportunities in order to generate innovative products (Figure 8).

The use of natural polymers in hydrogels manufacturing, especially on anindustrial scale, is associated with some drawbacks such as difficulties to ensure the reproducible properties from batch to batch, high costs, lower mechanical strength over time, variable stability, and high risks of microbial contamination for some finished products. A wide variety of natural polymers were tested to design SHs, but despite favorable effects on wound healing, some critical aspects were outlined which still need to be address for successful development of innovative formulations (Table 2).

The most used natural polymers for SHs formulation are presented below.

Dextran is an exopolysaccharide derived from the condensation of glucose (D-glucoses linked by α-(1→6) bonds, with possible branches of D-glucoses linked by α-(1→4), α-(1→3), or α-(1→2) bonds), obtained through fermentation of some species of lactic acid bacteria (*Leuconostoc mesenteroides*, *Weissella*, *Lactobacillus*, and *Streptococcus*) or by enzymatic polycondesation of glucose from sucrose via dextran saccharases. It is a neutral polysaccharide with good solubility in water, glycerol, and electrolyte solutions depending on its molecular weight and pH of media, and it is biocompatible, biodegradable, and stable in topical formulations. Moreover, dextran is nontoxic, being included in the GRAS list of substances since 1975 [133]. Food and Drug Administration (FDA) mentioned dextran on IIG as being accepted in formulation of products for topical and intravenous routes [134].

In wound healing, dextran has beneficial effects due its antioxidant, antimicrobial, and immunomodulatory ability, as well as promoting of angiogenesis in damaged tissue, properties which appear to vary with its molecular weight. For example, 40 kDa dextran from *Leuconostoc mesenteroides* showed immunomodulatory ability much better than 10 kDa and 147 kDa dextran. More exactly, with 40 kDa dextran, a 50% increasing murine macrophages proliferation and a 40% decreasing in cell release rate of nitric oxide were noted. Moreover, 40 kDa dextran showed the highest radical scavenging activity, with 70% inhibition of lipid peroxidase [135].

For dextran-based hydrogels formulation, both physical and chemical cross-linking methods are suitable. The characteristics of dextran-based hydrogels (as rheological behavior, mechanical strength, release of APIs embedded in their 3D matrix, stimuli-responsiveness) depend on the type of cross-linking method as well as on cross-linkers concentration. Using physical cross-linking methods, self-healing hydrogels with swelling behavior responsive to pH variations and promising controlled release of APIs were obtained. Chemical cross-linking based on thermal-gelation, radiation, or addition of divalent cations (Zn^2+,^ Mg^2+,^ Ca^2+^) also leads to versatile hydrogel with superior stability over time [136].

Due their ability of modulating rheological behavior of 3D matrices, dextran and its derivatives (methacrylated, aminated, oxidized) were used to develop non-Newtonian pseudo plastic hydrogels, whose viscosity decrease when increasing the shear rate. Epichlorohydrin and basic amino acids were successfully used for cross-linking pH-responsive dextran-based hydrogels. Thus, dextran-allyl isocyanate-ethylamine hydrogel demonstrated beneficial effects for promoting neovascularization and total skin regeneration (with hair follicles and sebaceous glands) in murine burn wound model [137]. A dextran/HA-based hydrogel showed a sustained release of sanguinarine embedded in the 3D matrix, stimulated fibroblast cell proliferation, and enhanced the healing of infected burn wounds [138]. Oxidized dextran and methacrylated gelatin were also used to formulate hydrogels for embedding black phosphorus nanosheets and zinc oxide NPs, which proved immunomodulatory effects on macrophage with enhanced secretion of anti-inflammatory factors [139]. A dextran/bacterial cellulose-based hydrogel showed superior cell proliferation and skin regeneration on an in vivo wound model. Moreover, addition of dextran in the hydrogel formulation endowed the final product with non-cytotoxicity compared with unmodified cellulose [140]. Other information related to use of dextran and dextran derivatives for SHs formulation are presented in Table 3.

Chitosan (CS) is a cationic polysaccharide with semicrystalline structure resulting from repeated units of N-acetyl-D-glucose amine and D-glucose amine, linked by β-(1-4) bonds. It was discovered more than 100 years ago when it was obtained by chemical or enzymatic deacetylation of chitin harvested from different sources [145]. The sources of chitin are varied, being found both in animal and vegetable, as insects (cuticle, cocoon), crustaceae (exoskeleton of crabs, shrimps), diatomae (*Thalassiosira fluviatilis*, *Algae*), and fungi (*Mucorrouxi*, *Aspergillis nidulans*) [146]. In 1970, after a long period of extensive research and scientific doubts, the foundations were laid for the practical use of CS. Nowadays, CS is one of the most used polymers based on its characteristics and biological properties such as excellent biocompatibility, biodegradability, non-toxicity, antioxidant, antimicrobial, and hemostatic effects, and mucoadhesivity [147]. Based on primary amino groups, CS has a pKa ~ 6.5 and initiates protonation in a neutral environment, which is accentuated as the pH decreases and the degree of deacetylation increases. Thus, CS becomes soluble in aqueous solutions and has bioadhesion capacity by binding to electro-negatively charged surfaces (for example, it can interact with sialic acid, a glycoprotein presents in mucus) [146].

CS is used for a wide area of pharmaceutical applications: mucoadhesive tablets, scaffolds for controlled release of drugs, growth factors, gels, films, beads, and liposomes. The lack of toxicity (LD_50_ (mouse, oral) > 16 g/kg) and structural similarity with the proteoglycans of the extracellular matrix make CS very attractive for medical applications, including wound care [148,149]. Due to its positive charge, CS interacts with the negative ions of red blood cells and platelets, facilitating coagulation and thus homeostasis restoration, which represents a challenge in wound management [150,151].

Through the action of lysozyme from the damaged tissues, CS splits into oligomers, which stimulate the synthesis and orientation of collagen fibers to restore the components of the extracellular matrix [152]. CS also stimulates the migration of inflammatory cells, macrophages, and fibroblasts, thus accelerating the inflammatory process [153]. In this way, the inflammatory phase is reduced, and the proliferative phase starts earlier in wound healing. In addition, there is a large quantity of data which sustain the antimicrobial activity of CS over a wide variety of microorganisms involved in wound colonization, such as *Escherichia coli*, *Staphylococcus aureus*, *methicillin resistant Staphylococcus aureus*, *Salmonella typhimurium*, *Pseudomonas aeruginosa*, *Candida albicans*, and *Enterococcus faecalis* [154,155]. This effect is due to the positively charged amino groups of CS that bind to the negatively charged cell surface, disrupt the cell membrane, and cause cell death by inducing leakage of intracellular components. The inhibition of genetic material synthesis by binding to the microbial DNA is also possible due to the presence of the protonated amino groups [154]

The effectiveness of CS in the treatment of wounds depends on its physico-chemical properties: degree of deacetylation, molecular weight, viscosity, and acetyl group distribution [146,156]. These characteristics vary from batch to batch, depending on the source of chitin, the period of the year of harvesting for the same source of chitin, and technological process for converting chitin into CS. There are several types of CS for cosmetic, pharmaceutical, and food industries, with molecular weights in the range of 10^4^–10^6^ Da and up to 95% deacetylation degree. The degree of deacetylation modulates is directly proportional to the antimicrobial action. The greater the degree of deacetylation, the greater the number of free amino groups available for protonation and the positively charge increases [154]. In addition, the molecular weight influences the ability of microbial inhibition, with CS with low and medium molecular weight penetrating the bacterial membrane more easily than CS with high molecular weight [146].

To increase the solubility, enhance the antimicrobial effects, and modulate the hemostatic and bioadhesive properties of CS, different physical, chemical, and enzymatic methods such as quaternization, thiolation, sulfation, copolymerization, and carboxymethylation have been reported [157]. Other information related to the use of CS and CS derivatives for SHs formulation are presented in Table 4.

Alginate is an anionic natural copolymer, composed of blocks of guluronate and mannuronate, obtained by alkaline extraction from brown algaes (*Laminaria* spp., *Ascophyllumnodosum*, *Macrocysti spyrifera*) or by microbial synthesis (*Azotobacter*, *Pseudomonas*). It is characterized by several properties, such as biocompatibility, low toxicity, economical affordable, and ease of gelation, which makes alginate attractive for biomedical applications such as tissue engineering, drug delivery, biosensing, and wound healing [164,165].

Alginate salts (ammonium, potassium, calcium, sodium) and alginate derivatives (propylene-glycol alginate) are GRAS listed for 50 years, being considered safe for use in food industry in Europe. The FDA has included alginates on the IIG list for products administrated via topical and oral routes [133,134].

Several studies confirm the cytoprotective capacity of alginates due their antioxidant effects and immunoregulatory activity. Moreover, alginates oligosaccharides (guluronate and mannuronate) have proved anti-inflammatory effects through interfering with the production of nitric oxide, prostaglandins, and pro-inflammatory cytokines. The proliferation of human keratinocytes and endothelial cells was also attributed to alginates, especially due the presence of guluronic acid at the end of the backbone chain. These effects are related to molecular height, alginate sources, guluronate: mannuroanate ratio, as well as purification and extraction technologies. The alginate with the highest molecular weight (MW 38,000) and higher mannuronate: guluronate ratio (2.24) exhibited a higher effect on the stimulation of the secretion of tumor necrosis factor-α (TNF-α) in murine macrophage cell line [166,167].

Topical alginate-based products (dressings, films, foams, fibers, and hydrogels) may absorb up to 20 times their weight in moisture, promoting tissue regeneration of highly exudative wounds such as venous ulcers, pressure ulcers, and infected and large surgical wounds. The combination of alginates with different natural and synthetic polymers leads to biomimetic and biodegradable hydrogels, which could be used to achieve different targets (antibiotics and protein delivery, cell culture). Other information related to the use of alginate and alginate derivatives for SHs formulation are presented in Table 5.

Hyaluronic acid (HA) is a natural anionic glycosaminoglycan, consisting of D-glucuronic acid and D-N-acetylglucosamine units. Unlike other glycosaminoglycans (heparin, chondroitin sulfate, dermatan sulfate, keratin), HA does not contain sulfur, it is not synthesized by the action of the enzymes of the Golgi apparatus on proteins, and may have higher molecular weights up to 10^5^ kDa (compared to maximum 50 kDa for other glycosaminoglycans) [173]. HA is widely distributed in nature, being found in the epithelial, connective, and nervous tissue of vertebrates, but also in the cells of microorganisms (*Streptococcus equi*, *Streptococcus zooepidermicus*, *Streptococcus equisimilis*, *Streptococcus pyogenes*, *Streptococcus uberis*, *Pasteurella multocida*, *Cryptococcus neoformans*) [85]. The human body contains approximately 215 mg HA/kg, and 50% is found in skin, dermis, and epidermis. The synthesis of HA is enzymatic controlled by hyaluronan synthetases (HASs) (HAS1, HAS2, HAS3, located on the inner surface of the cellular membrane), while hyaluronidase and oxidative stress are implicated in its degradation. The turnover is very fast (5 g/day), and the half-life is less than 24h [174].

Currently, HA is a key molecule in the medical, pharmaceutical, nutrition, and cosmetic industries and, as result, the researchers are looking for new synthesis routes and optimization of production biotechnologies or structural modulation in order to obtain new derivatives with improved features [148,175,176].

In wound healing, HA is involved in each phase, the effects being dependent to its molecular weight. For example, HA with a high molecular weight exhibits anti-inflammatory effects, while HA with a low molecular weight is responsible for mediating inflammation, with both possessing scavenging properties against oxygen free radicals [175,177,178].

In the hemostasis phase, white blood cells produce a significant amount of high molecular weight HA (4 × 10^2^–2 × 10^4^ kDa), which by interaction with fibrinogen forms temporary white platelets. Due to the hydrophilic character of HA, water is attracted to the area of the thrombus and edematization of the area is initiated, with the temporary blocking of the penetration of immune cells into the injured area [176]. In the inflammatory phase, HA with high molecular weight is degraded into fragments smaller than 120 kDa, with immunostimulatory properties and a proangiogenesis effect. These products stimulate the production of cytokines (IL-1 and IL-8, TNF-α) and, consequently, the infiltration, activation, and maturation of immune cells are accelerated [174,179,180]. During the proliferation phase, HA with low molecular weight is degraded to oligomers, which will interact with the CD44 and RHAMM receptors. As a result, keratinocytes, fibroblasts, and endothelial cells mature and activate, which translates into inflammation reduction, support for angiogenesis, stimulation of granulation tissue, and synthesis of type III collagen (immature collagen) [49,176,181]. Finally, the oligomers determine the synthesis of matrix-metalloproteinase and TNF-β. These mediators are necessary for remodeling the extracellular matrix through the differentiation of fibroblasts into myofibroblasts and the maturation of collagen [176,182].

Based on physical and biological characteristics, HA and its derivatives are used in the manufacturing of wound care such as hydrogels, dressings, foams, nanofibers, and sponges.

Through etherification or esterification of the hydroxyl and carboxyl groups with different chemical compounds (hydrazide, phenylboronic acid, polyphenols), increasing performance of 3D matrices was obtained, without the necessity of blending with other polymers [180]. Other information related to the use of HA and HA derivatives for SHs formulation are presented in Table 6.

##### Biological-like Polymers

Self-assembling peptides (SAPs)

Peptides are comprised from aminoacid residues linked through peptide bonds, with a molecular weight of less than 10,000 Da. Peptides are ubiquitously present in both vegetal and animal regna and are essential for life cycles. Every physio/pathological process is regulated by various types of peptides with one or more functions. So, there are antibiotic peptides which are part of natural immune systems of prokaryotes and eukaryotes, cancer/anticancer peptides, immune/inflammatory peptides, endocrine peptides, gastrointestinal peptides, cardiovascular peptides, renal peptides, respiratory peptides, opioid peptides, and blood–brain peptides. In organisms, peptides are synthesized through ribosomal translation (antibiotics in microorganisms–microcines and bacteriocines), hormones and other signaling molecules (in the human body), or by enzymatic processes (glutathione) [185].

The use of peptides in the formulation of a 3D matrix is relatively recent and is based on the intra- and intermolecular self-assembling ability of aromatic short peptides, biomimetic peptides, amphiphilic peptides, and dendrimers. In the assembling process, super molecular structures are formed, such as spherical or worm-like structures, bilayer micelles, high ratio aspect nanofibers, twisted ribbons, nanotubes, or flat forms, through non-covalent interactions (hydrogen bonds, hydrophobic and electrostatic interactions, van der Waals forces). The resulting structures are highly biocompatible and may be used in tissues regeneration, scaffolds for drugs carriers and controlled release, and modulation of APIs to overcome adverse effects and enhance their stability [186].

The discovery of the first SAP, coded Ac-(AEAEAKAK)2-CONH_2_ (A-alanine, E-glutamic acid, K-lysine), in a yeast protein zuotin in 1990 was regarded as a big innovation and a valuable tool to design new materials for a wide variety of applications [186]. The chemical composition of SAPs consistsof alternating hydrophobic aminoacids (alanine, valine, leucine, isoleucine, and phenylalanine) with hydrophilic aminoacids (lysine, arginine, histidine, aspartic acids, and glutamic acids), having very well-defined arrangements of negative and positive charges [187].

Hydrogels based on SAPs are sensible to multiples stimuli such as pH, temperature, ionic strength, and redox activity as a result of charging the amino and carboxyl groups on the peptide backbones [186]. Due the resembling natural extracellular matrix, these formulations are suitable for adhesion, migration, differentiation, and proliferation of cells involved in wound healing.

The weak bonds within an SAP 3D structure could be easily modulated to achieve targeted SAP performance, such as mimicking extracellular matrices, recognition of ligandreceptors, and modification of their structural architecture. For example, the SAP solution undergoes phase transition to a gel state under pH and ionic strength variations. In this way, when are applied on damaged tissues with irregular shapes and depths, an intimate contact is assured, the wound is filled with the product, and so the hemostasis is fast and the healing is accelerated.

The temperature induces different 3D organizations of the peptide aggregates which influence the water uptake and mechanical strengths of hydrogels. For instance, amyloid beta peptide (C16-KKFFVLK) exhibits different self-arrangements at 20 °C (helical ribbons and nanotubes) compared to its exposure to 55 °C (twisted tapes). In addition, peptide RADA 16-I maintains beta sheets assembling through 25 °C to 70 °C, but small-sized globular aggregates were obtained at much higher temperatures [186,187,188].

Targeting microorganism resistance to antibiotherapy, SAPs nanofibers with antimicrobial effects have been developed [189]. It was shown that diphenylalanine-SAP inhibits bacterial growth through cell membrane disruption, depolarization, and enhanced permeation resulting in elevated levels of ROS [190]. The addition of short SAPs to natural or synthetic polymers allows the design of SHs with improved benefits on wound healing [181]. Arg-Gly-Asp-(RGD), cytosine-guanine-adenine (CAG), Arg-Glu-Asp-Va (REDV), and Tyr-Ile-Gly-Ser-Arg (YIGSR) are short SAPs used to activate the physiological cascade of wound healing because they are recognized by integrin receptors (RGD) or endothelial cells and promote cell migration, granulation tissue formation, selective cell adhesion, and neoangiogenesis in damaged tissues [191].

PuraStat^®^, PuraBond^®^, PuraDerm^®^, and PuraGel^®^ are commercial products manufactured by 3-D Matrix Europe SAS, France, based on a syringe prefilled acid solution (pH 2) of self-assembling RADA16 peptide (which is composed of alternating positively charged arginine (R), hydrophobic alanine (A), and negatively charged aspartic acid (D), that repeat periodically throughout the composition). The acid peptide solution is neutralized in contact with blood or physiological fluids and through exposure to ionic species (Na^+^, K^+^), resulting in clear hydrogels that mimic the extracellular matrix with a fast hemostatic effect, which serve as a mechanical barrier for exposed tissue and prevent the formation of post-operative adhesions. The viscosity of the SAP solutions allows easy application, using either a standard applicator, endoscopic catheter, or laparoscopic applicator, making the products applicable to different kinds of injuries such as surgical wounds, nasal bleedings, deep wounds (pressure ulcers, diabetic ulcers), and dental surgery [68,186,192,193,194].

Deoxyribonucleic Acid

Exceeding the initial focus on genetic information transmissions, synthetic deoxyribonucleic acid (DNA) has been extensively investigated for new drug discovery, modified release matrices, diagnostics tools, biomolecular therapies (including vaccines, enzymes, and hormones), and in vivo metabolism modulation up to creating an artificial living organism, as interest areas for new generation medical fields. Based on the chemical features of DNA and nucleobases (adenine, cytosine, guanine, and tyrosine), synthetic DNA is suitable for molecular and topological editing with specialized software (caDNAno, Tiamat, Nupack, MagicDNA) in order to reproducibly and predictably design complex dynamic or static structures [195].

Tetrahedral framework nucleic acids (a static DNA tetrahedral nanostructure) were tested on multiple in vivo wounds model and proved significant anti-inflammatory and antioxidant effects, preventing tissue destruction and sustaining regeneration on skin and acute kidney injury, as well as in osteoarthritis [195,196].

Dynamic synthetic DNA-based nanostructures react with different types of biomolecules (nucleic acid, proteins) and are sensible to environment variations (pH, temperature), which make them attractive for tissue engineering. A cost-effective self-assembled DNA-based hydrogel was developed through electrostatic interactions and hydrogen bonds between DNA structure and tetrakis(hydroxymethyl) phosphoniumsulfate (THPS) molecule. The hydrogel exhibited self-healing abilities, good cytocompatibility, prevented wound microbial colonization through release of THPS, and enhanced the healing process. In another study, DNA units, polyacrylamide, and L-ascorbic acid 2-phosphate were dynamically cross-linked through hydrogen bonds in 3D hydrogel, which mimics extracellular matrices. The hydrogel was loaded with borneol to relieve pain and itching and was proved to initiate the phosphatidylinositol 3′-kinase signaling pathway, which promoted cell proliferation and regeneration in burn wounds [197].

#### 4.4.2. Synthetic Polymers

**Figure 9:**
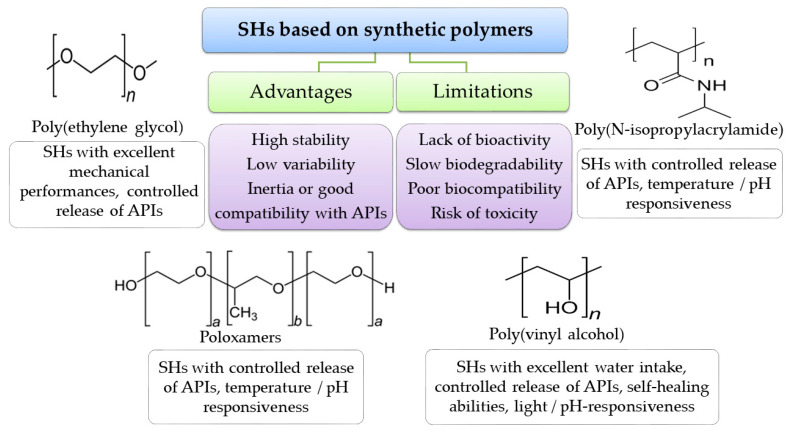
Synthetic polymers used in SHs formulation for wound healing management.

Synthetic polymers (as PVA, PEG, ploy(acrylamides), poloxamers) offer a more reproducible physical and chemical characteristics from batch to batch of both raw materials and hydrogels, higher flexibility and mechanical strength, very good stability over time, compatibility or inertia for a wide range of APIs, and often allow higher water intake than natural-based-hydrogels. However, some drawbacks, such as poor biocompatibility, high risk of toxicity, slow biodegradability, and lack of bioactive effects, are associated with synthetic polymers (Figure 9).

The combination of natural and synthetic polymers could improve the performance of hydrogels. So, the biocompatibility and bioactivity of natural structures are sustained by the robust structures of synthetic components, which result in improved therapeutic benefits, costs optimization, and support of the industrial implementation of innovative SHs manufacturing for biomedical applications. The most used synthetic polymers for SHs formulation are presented below.

Poly(ethylene glycol) (PEG) is a stable hydrophilic polymer derived from petroleum or synthesized from ethylene oxide and water in both acidic and alkaline conditions. Depending on reactants ratio and conditions, a wide variety of products with different molecular weights (3 × 10^2^–10^7^ g/mol) and different geometries of polymer chains (star, comb, branched) could be manufacturing. It is interesting to note that although the physical characteristics (density, viscosity, aggregation state) depend on structural properties, all PEGs are chemically identical. The presence of hydroxyl groups of the end of the backbones endow the PEG with the ability of attaching to multiple reactive functional groups (methoxy, amino, thiol, vinyl sulfone, azide, acetylene, and acrylate), which improve its performance. Moreover, PEG may be attached to large chemical entities such as proteins, polysaccharides, oligonucleotides, or to small molecules such as mannose or folate to improve biodegradability and biocompatibility [198].

PEGs are considered as nontoxic, non-immunogenic, and nonirritant substances, being included in the IIG database for parenteral, oral, and topical pharmaceutical products [134]. For pharmaceutical applications, PEGs may be used as ointment/suppository bases, plasticizers, solvents, emulsifiers, surfactants, tablets, and capsule lubricants. In addition, branched PEGs derivatives were investigated for their applicability in the development of biodegradable controlled-release matrices, regenerative medicine wound sealing, and wound healing. Aqueous solutions of higher-molecular-weight PEGs may form flexible, highly hydrophilic gels suitable for wound care using different cross-linking methods (radiation, free radical polymerization, condensation, click chemistry, enzymatic reactions, etc.) [199,200].

Poly(vinyl alcohol) (PVA) is a widely used highly water-soluble polymer obtained through free-radical polymerization of vinyl acetate, followed by alkaline hydrolysis of acetate groups. PVA has been shown to be chemically stable in a wide range of pH and temperatures, being suitable for various chemical and technological modulations. Because of its semicrystalline structure, it allows both the vital molecules (oxygen, glucose, and aminoacids) and metabolism products to cross the cellular membrane. In addition, PVA is biocompatible, biodegradable, and bioinert, being included in the IIG database of solutions, suspensions, creams, aerosols, foams, tablets, and capsules for topical and oral routes [134].

The physical, chemical, and biological features of PVA support its use for a wide range of biomedical applications (implantable medical devices, tissue regeneration–artificial cartilage and meniscus, contact lens, wound dressings, artificial tears). PVA-based hydrogels proved excellent mechanical strengths, bioadhesion, and high water absorption uptake.

In order to design 3D networks using PVA, both a chemical and physical method may be used. When applying repeated freezing–thawing cycles on PVA solutions, without addition of external cross-linkers, clear, pure hydrogels with enhanced biological aging resistance were obtained. The size and conformation of the hydrogel matrix are influenced by the solvents (glycerol, ethylene glycol), molecular weight, and concentrations of the PVA solutions. These characteristics are very important because they influence the performance of the hydrogel on drug delivery, moisturizing the wound bed, and the hydrogel flexibility for damaged tissue protection. It was noted that the water uptake enriched the hydrogels with higher mechanical strength, while using ethylenglycol increased the toughness of the products [201]. PVA-based SHs with versatile features such as self-healing, stimuli responsiveness, and an improved controlled drug release profile were developed.

Poloxamers is a generic name for a group of nonionic water-soluble copolymers with amphiphilic structure, due their central hydrophobic component (polyoxypropylene) and peripheral hydrophilic chains (polyoxyethylene), HO(C_2_H_4_O)_x_(C_3_H_6_O)_y_ (C_2_H_4_O)_x_H. Depending on the molecular weight and hydrophobic: hydrophilic ratio of the polymers, the solution of poloxamers exhibited reversible temperature-depended phase transitions with self-assembling gelation. At low temperatures and at concentrated solutions (above critical micellar concentrations), the poloxamers are liquids. Upon skin contact, the polyoxyproylene core starts the dehydration and micellization process, while the polyoxyethylene blocks remain soluble.

Poloxamers are widely used in the cosmetic and pharmaceutical industry as surfactants, emulsifiers, solubility enhancers, stabilizers, viscosifiers, cell culture media, drugs carriers, and in nanofiber synthesis [202,203]. These copolymers are included by the FDA in the IIG list, accepted for intravenous injections, inhalations, ophthalmic preparations, oral powders, solutions, suspensions, and syrups, and topical preparations [134].

Poly(N-isopropylacrylamide) (PNIPA) is a thermoresponsive water-soluble polymer synthesized through free radical polymerization of N-isopropyl acrylamide. Depending on the molecular weight and polymer dispersity, PNIPA undergoes reversible phase transition from a solution at lower temperatures to gel a state at near-body temperature (32 °C ± 5 °C), which makes it attractive for medical applications such as wound dressings, tissue engineering (artificial muscles), scaffolds for controlled release of pH-sensitive drugs, macro and microgels formulations, cell culture, and biosensors.

Functionalization of the end-chain of PNIPA with different moieties, such as pyrenil, azobenzene, thiol, and azide, endows the polymer with tunable properties, such as a modified low critical solution temperature, pH-sensitivity, and temperature responsivity [204,205,206].

In wound healing, the PNIPA solution with different APIs added is suitable to readily apply on damaged skin and use the body temperature to convert it into a 3D matrix with hemostatic activity. The adjustable geometries of the hydrogel matrix allow an intimate contact with the wound bed, helping to create a proper environment for proliferation and differentiation of cells involved in healing process [207].

## 5. Conclusions

The prevalence of acute and chronic wounds is increasing every year because of the aging population and the growing incidence of wound-related comorbidities, and remains a continuous concern of health systems worldwide. Overtime, medical specialists have developed and implemented various protocols and tools to overcome misinterpretation in wound assessment, which lead to impaired healing processes and complications. Choosing the appropriate topical approach has proved to be crucial for the entire output of the treatment.

Hydrogels have passed the test of time, being used for more than 120 years in wound healing. Because of their undeniable benefits in the healing process, hydrogels constantly have attracted the interest of researchers in the medical and pharmaceutical area and sustained efforts are made to improve and to innovate the performances of hydrogels and to widen their biomedical applications. SHs is a large category of versatile 3D matrices which has made it possible to design intelligent products with the target of overcoming the limitation of traditional hydrogels and properly responding to the increasing prevalence of wounds with impaired healing.

Althoughimpressive research work is ongoing in the SHs field and various SHs have been proved to exhibit innovative properties, some drawbacks are still to be investigated.

The controlled release of APIs is one of the top interest areas in SHs development. Despite this, in vitro release tests showed reproducible kinetics, and in vivo assessment could be affected by specific wound environment composition and irregular vascularization, which could lead to over-release with potential toxic effects or, on the contrary, to a decreased release rate which, especially in the case of antibiotics, leads to complications due to under-dosing. A future target of research could be the inclusion of biosensors in 3D matrices, where online data regarding wound bed biochemistry and the command and controlled release of the required number of APIs could be collected.

Reversible stimuli-responsive hydrogels are very attractive due their ability to overcome exposure to a wide variety of external or internal stressors (mechanical, thermal, chemical). Thus, the ability of hydrogels to undergo structure breakage when they are passed through a syringe needle and to quickly restore their matrix is an excellent feature for products designed to be injected in deep, irregular wounds. These hydrogels are more commonly based on physical interactions, which are less stable than covalently cross-linked networks. This is translated into low stability over time, which is a serious limitation of industrial-scale applicability. The dynamic covalent interactions and adequate functionalization of polymers may be successfully used to address this issue.

The biodegradability and non-cytotoxicity of SHs are widely discussed, but the behavior of the degradation products in human body is still unclear and not fully evaluated (e.g., PNIPA-based SHs may generate carcinogen moieties). Further investigations might comprehensively address this issue and the degradation pathways and resulting compounds should be carefully documented. Moreover, the SHs showed promising healing effects on various in vivo wound models, but clinical trials were not yet conducted in order to evaluate the human safety of the finished products, to establish the optimal method of application and dosage, and to assess the risk/benefits balance, in comparison with commercially available alternatives.

## Figures and Tables

**Table 1:** The relevant particularities of traditional hydrogels versus SHs, designed for wound healing.

Traditional Hydrogels	SHs	**References**
Protection of the damaged area to external factors	Enhanced protection of the damaged area due the self-adapting ability	[36,43]
Provide moisture of the wound bed depending on hydrogel composition without regarding on wound conditions (dry, wet)	Dynamic and interactive control of moisture, based on responsive behavior to wound condition (exudate, pH) or external triggers (pressure, light)	[42,62]
Frequent changing during treatment	The need of dressing changes may be reduced and adapted to patient and wound particularities	[12,61]
Strong adhesive properties with discomfort and secondary injuries when they are removed or lack of adhesive properties with inadequate contact with the damaged tissue	Excellent adhesive properties with intimate contact to the wound bed and easiness remove without damages or discomfort for patient	[63,64,65]
Low or no protection against microbial contamination	Efficient protection to microorganism growth through polymers characteristics, cross-linking methods, and manufacturing design	[60,64]
No effect on physiological mechanisms of healing process	Promote, accelerate, and modulate the endogenous mediators on wound site	[35,46]
A limited number of APIs (most water-stable) could be embedded into 3D matrix	A wide variety of APIs (chemical and bioactive molecules) could be embedded using versatile carriers (vesicles, liposomes, niosomes, nanoparticles)	[45,59]
Passive release of APIs.	Controllable release of API through endogenous or exogenous stimuli, depending on wound changes	[46,66]
No possibility to evaluate the wound condition during treatment	Possibilities to real-time monitor the healing of wound	[59,67]
Low cost of production, simple design, robust manufacturing at industrial scale	Challenging manufacturing process, difficulties to reproduce the quality attributes from batch to batch	[61,68]
Safe raw materials with well-established characteristics for human use	New synthetized raw materials or modified from traditional sources which may raise stability and safety issues	[39,55]
Simple to use, easy to apply	Requires training of the patient and/or medical staff	[38,42]

**Table 2:** Challenges innatural polymers in the development of SHs for wound healing.

Polymer	Advantages	Challenges	Polymer-Based SHs (e.g.)	References
Gelatin	Haemostatic effect, promote migration of fibroblasts on injured area	Hypersensitivity reactions; 3D structure is destroyed through bacterial contamination	Antibacterial injectable self-healing hydrogel based on gelatine and poly(ethylene glicol) bis (benzandehide) loaded with clindamycin; Antibacterial self-healing hydrogel based on gelatine methacrylate, adenine acrylate and copper(II) chloride	[117,118,119]
Collagen	Haemostatic effect, promote cell migration, proliferation and differentiation; stimulate formation of granulation tissue and synthesis of ECM with reduced scars; anti-inflammatory and proangiogenic effects	High cost, enzymatic degradation, poor elasticity; no gelling properties (requires additional polymers)	pH- and ROS-responsive hydrogel based on recombined collagen and HA with controlled release of APIs; Collagen/xanthan gum-based hydrogels with antibacterial effect, and controlled release of ketorolac and methylene blue	[117,120,121]
Guaran	Haemostatic effects; lack of toxicity	Risk of bacterial contamination and of depolymerization	Self-healing injectable hydrogel based on oxidized quaternized guaran/carboxymethyl CS with hemostatic and antibacterial effects	[122]
Elastin	Chemotaxis to neutrophils, monocytes, macrophages, fibroblasts and endothelial cells, protease production (upregulation of matrix metalloprotease), mimics ECM	Insoluble in water, rapid degradation, low mechanical stability; cross-linking methods affect the elastin performance	Thermoresponsive hydrogels with shape-memory ability based on elastin and elastin-like polypeptides	[123,124,125,126]
Silk	Stimulates cell migration, proliferation, and collagen production, wound remodeling	Insoluble in water, immunogenic and inflammatory effects (due to sericin content), high cost for sericin free silk	Self-healing hydrogels based on silk-fibroin and β-cyclodextrin; Desferrioxamine-loaded injectable silk nanofibers hydrogels for diabetic wounds	[127,128,129,130]
Cellulose	Lack of toxicity, low cost	Insoluble in water or other common solvents; no bioactivity; allergic reactions	Magnetic responsive hydrogel based on cellulose and β-cyclodextrin to control drug release by swelling behavior	[131,132]

**Table 3:** SHs based on dextran and dextran derivatives for wound healing.

Polymers	APIs	SHs Features	References
Oxidized dextran/quaternized CS	-	Stimulation of myoblast proliferation under electrical field; injectability	[141]
Oxidized dextran	Sulfadiazine and tobramycin	pH-responsive injectable hydrogel with controlled release of APIs	[142]
Oxidized dextran/peptide DP7 (VQWRIRVAVIRK)	Ceftazidime	pH-responsive hydrogel with scarless healing of multidrug-resistant infected wounds	[143]
Oxidized dextran/aminated gelatin	ZnO, paeoniflorin, norfloxacin	ROS-responsive hydrogel	[144]

**Table 4:** SHs based on CS and CS derivatives for wound healing.

Polymers	APIs	SHs Features	References
CS/oxidized konjac glucomannan	Silver (nanoparticles)	Self-healing, self-adapting	[71]
Oxidized CS/bacterial cellulose	-	Self-healing	[158]
N,O-carboxymethyl CS/oxidized dextran	-	Self- healing, injectability	[159]
Quaternized CS/oxidized dextran	Tobramycin	Self-healing	[160]
Carboxymethyl CS/oxidized quaternized guaran	-	Self-healing	[122]
CS/oxidized CS	Fusidic acid, alantoin, coenzyme Q10	Self-healing, self-adapting	[161]
CS/polyvinylpyrrolidone/alginate/poly(ε-caprolactone)	-	Thermo-responsive	[162]
CS/poly(aspartic acid)	Amoxicilin	pH/thermo-responsive	[163]

**Table 5:** SHs based on alginate and alginate derivatives for wound healing.

Polymers	APIs	SHs Features	References
Alginate	Amikacin, naproxen	pH/ROS-responsive	[109]
Alginate/HA	Doxycycline	ROS–responsive	[60]
Oxidized alginate/gelatin	Ishophloroglucin A	Dynamic mechanical properties	[168]
Alginate/polyacrylamide	-	pH-responsive (color change)	[169]
Alginate/pluronic F127	Vascular endothelial growth factor	Thermo-responsive	[170]
Alginate/polycaprolactone	Melatonin	Controlled release of APIs	[171]
Alginate/HA	Platelet rich plasma	ROS/glucose-responsive, self-healing, injectability	[172]

**Table 6:** SHs based on HA and HA derivatives for wound healing.

Polymers	APIs	SHs Features	References
Phenylboronic acid-modified HA	Tannic acid–silver (nanopartices)	pH/ROS-responsive, self-healing	[98]
Adipic acid dihydrazide grafted HA	Sisomicin sulfate	pH-responsive	[183]
Tannic acid grafted HA	Silver nanoclusters and deferoxamine	pH-responsive, injectability	[184]

## Data Availability

The data could be requested from authors.

## References

[1] Demidova-Rice T.N., Hamblin M.R., Herman I.M. (2012). Acute and Impaired Wound Healing: Pathophysiology and Current Methods for Drug Delivery, Part 1: Normal and Chronic Wounds: Biology, Causes, and Approaches to Care. Adv. Skin. Wound Care.

[2] Gethin G., McIntosh C., Cundell J. (2011). The Dissemination of Wound Management Guidelines: A National Survey. J. Wound Care.

[3] Zhu X., Olsson M.M., Bajpai R., Järbrink K., Tang W.E., Car J. (2022). Health-Related Quality of Life and Chronic Wound Characteristics among Patients with Chronic Wounds Treated in Primary Care: A Cross-Sectional Study in Singapore. Int. Wound J..

[4] Yao Z., Niu J., Cheng B. (2020). Prevalence of Chronic Skin Wounds and Their Risk Factors in an Inpatient Hospital Setting in Northern China. Adv. Skin Wound Care.

[5] Järbrink K., Ni G., Sönnergren H., Schmidtchen A., Pang C., Bajpai R., Car J. (2016). Prevalence and Incidence of Chronic Wounds and Related Complications: A Protocol for a Systematic Review. Syst. Rev..

[6] Eriksson E., Liu P.Y., Schultz G.S., Martins-Green M.M., Tanaka R., Weir D., Gould L.J., Armstrong D.G., Gibbons G.W., Wolcott R. (2022). Chronic Wounds: Treatment Consensus. Wound Repair Regen..

[7] Sen C.K. (2019). Human Wounds and Its Burden: An Updated Compendium of Estimates. Adv. Wound Care.

[8] Jupiter D.C., Thorud J.C., Buckley C.J., Shibuya N. (2016). The Impact of Foot Ulceration and Amputation on Mortality in Diabetic Patients. I: From Ulceration to Death, a Systematic Review. Int. Wound J..

[9] Cheun T.J., Jayakumar L., Sideman M.J., Ferrer L., Mitromaras C., Miserlis D., Davies M.G. (2020). Short-Term Contemporary Outcomes for Staged versus Primary Lower Limb Amputation in Diabetic Foot Disease. J. Vasc. Surg..

[10] Gushiken L.F.S., Beserra F.P., Bastos J.K., Jackson C.J., Pellizzon C.H. (2021). Cutaneous Wound Healing: An Update from Physiopathology to Current Therapies. Life.

[11] BuganzaTepole A., Kuhl E. (2013). Systems-Based Approaches toward Wound Healing. Pediatr. Res..

[12] Bowers S., Franco E. (2020). Chronic Wounds: Evaluation and Management. Am. Fam. Physician.

[13] Las Heras K., Igartua M., Santos-Vizcaino E., Hernandez R.M. (2020). Chronic Wounds: Current Status, Available Strategies and Emerging Therapeutic Solutions. J. Control. Release.

[14] Olsson M., Järbrink K., Divakar U., Bajpai R., Upton Z., Schmidtchen A., Car J. (2019). The Humanistic and Economic Burden of Chronic Wounds: A Systematic Review. Wound Repair Regen..

[15] Nagle S.M., Stevens K.A., Wilbraham S.C. (2023). Wound Assessment. StatPearls.

[16] Woo K.Y., Sibbald R.G. (2009). A Cross-Sectional Validation Study of Using NERDS and STONEES to Assess Bacterial Burden. Ostomy. Wound Manag..

[17] (2023). Calling on NERDS for Critically Colonized Wounds: Nursing. https://journals.lww.com/nursing/Citation/2007/05000/Calling_on_NERDS_for_critically_colonized_wounds.17.aspx.

[18] Wound Bed Preparation: Employing the TIME Acronym. https://www.independentnurse.co.uk/content/clinical/wound-bed-preparation-employing-the-time-acronym/.

[19] Nuutila K., Eriksson E. (2021). Moist Wound Healing with Commonly Available Dressings. Adv. Wound Care.

[20] Varma A., Warghane A., Dhiman N.K., Paserkar N., Upadhye V., Modi A., Saini R. (2023). The Role of Nanocomposites against Biofilm Infections in Humans. Front. Cell. Infect. Microbiol..

[21] Doughty D. (2005). Dressings and More: Guidelines for Topical Wound Management. Nurs. Clin. N. Am..

[22] Junker J.P.E., Kamel R.A., Caterson E.J., Eriksson E. (2013). Clinical Impact Upon Wound Healing and Inflammation in Moist, Wet, and Dry Environments. Adv. Wound Care.

[23] Wound-Management-Guidelines. https://www.bcpft.nhs.uk/documents/policies/w/1444-wound-management-guidelines/file.

[24] Ward J., Holden J., Grob M., Soldin M. (2019). Management of Wounds in the Community: Five Principles. Br. J. Community Nurs..

[25] Dowsett C., Swanson T., Karlsmark T. (2019). A Focus on the Triangle of Wound Assessment-Addressing the Gap Challenge and Identifying Suspected Biofilm in Clinical Practice. Clin. Pract..

[26] Gil S.B. (2020). Implementing the Triangle of Wound Assessment Framework to Transform the Care Pathway for Diabetic Foot Ulcers. J Wound Care.

[27] Dowsett C., Bain K., Bain M. (2020). Closing the Gap between the Evidence and Clinical Practice-a Consensus Report on Exudate Management. Wounds.

[28] Wound Assessment Tools: An Introduction to PUSH, NPUAP and Wagner. https://www.woundsource.com/blog/wound-assessment-tools-basic-introduction-push-npuap-and-wagner.

[29] Moore Z.E., Webster J. (2018). Dressings and Topical Agents for Preventing Pressure Ulcers. Cochrane Database Syst. Rev..

[30] Advanced Wound Care Market–Table of Content, List of Tables and List of Figures. https://www.factmr.com/report/advance-wound-care-market/toc.

[31] Derwin R., Patton D., Avsar P., Strapp H., Moore Z. (2022). The Impact of Topical Agents and Dressing on PH and Temperature on Wound Healing: A Systematic, Narrative Review. Int. Wound J..

[32] Barbu A., Neamtu B., Zăhan M., Iancu G.M., Bacila C., Mireșan V. (2021). Current Trends in Advanced Alginate-Based Wound Dressings for Chronic Wounds. J. Pers. Med..

[33] de Oliveira Ruiz P.B., Lima A.F.C. (2022). Average Direct Costs of Outpatient, Hospital, and Home Care Provided to Patients with Chronic Wounds. Rev. Esc. Enferm..

[34] Brumberg V., Astrelina T., Malivanova T., Samoilov A. (2021). Modern Wound Dressings: Hydrogel Dressings. Biomedicines.

[35] Ferreira N.N., Ferreira L.M.B., Cardoso V.M.O., Boni F.I., Souza A.L.R., Gremião M.P.D. (2018). Recent Advances in Smart Hydrogels for Biomedical Applications: From Self-Assembly to Functional Approaches. Eur. Polym. J..

[36] El-Husseiny H.M., Mady E.A., Hamabe L., Abugomaa A., Shimada K., Yoshida T., Tanaka T., Yokoi A., Elbadawy M., Tanaka R. (2022). Smart/Stimuli-Responsive Hydrogels: Cutting-Edge Platforms for Tissue Engineering and Other Biomedical Applications. Mater. Today Bio..

[37] Thakur S., Thakur V.K., Arotiba O.A., Thakur V.K., Thakur M.K. (2018). History, Classification, Properties and Application of Hydrogels: An Overview. Hydrogels.

[38] Hu C., Yang L., Wang Y. (2022). Recent Advances in Smart-Responsive Hydrogels for Tissue Repairing. MedComm.–Biomater. Appl..

[39] Li Z., Zhou Y., Li T., Zhang J., Tian H. (2022). Stimuli-Responsive Hydrogels: Fabrication and Biomedical Applications. VIEW.

[40] Stan D., Tanase C., Avram M., Apetrei R., Mincu N.-B., Mateescu A.L., Stan D. (2021). Wound Healing Applications of Creams and “Smart” Hydrogels. Exp. Dermatol..

[41] Parhi R. (2017). Cross-Linked Hydrogel for Pharmaceutical Applications: A Review. Adv. Pharm. Bull..

[42] Bahram M., Mohseni N., Moghtader M. (2016). An Introduction to Hydrogels and Some Recent Applications. Emerging Concepts in Analysis and Applications of Hydrogels.

[43] Xiang J., Shen L., Hong Y. (2020). Status and Future Scope of Hydrogels in Wound Healing: Synthesis, Materials and Evaluation. Eur. Polym. J..

[44] Liang Y., He J., Guo B. (2021). Functional Hydrogels as Wound Dressing to Enhance Wound Healing. ACS Nano.

[45] Ghasemiyeh P., Mohammadi-Samani S. (2019). Hydrogels as Drug Delivery Systems; Pros and Cons. Trends Pharm. Sci..

[46] Kaith B.S., Singh A., Sharma A.K., Sud D. (2021). Hydrogels: Synthesis, Classification, Properties and Potential Applications—A Brief Review. J Polym Environ..

[47] Baby D.K. (2020). Rheology of Hydrogels. Rheology of Polymer Blends and Nanocomposites.

[48] Bao Z., Xian C., Yuan Q., Liu G., Wu J. (2019). Natural Polymer-Based Hydrogels with Enhanced Mechanical Performances: Preparation, Structure, and Property. Adv. Healthc. Mater..

[49] Li S., Pei M., Wan T., Yang H., Gu S., Tao Y., Liu X., Zhou Y., Xu W., Xiao P. (2020). Self-Healing Hyaluronic Acid Hydrogels Based on Dynamic Schiff Base Linkages as Biomaterials. Carbohydr. Polym..

[50] da Silva C.M., da Silva D.L., Modolo L.V., Alves R.B., de Resende M.A., Martins C.V.B., de Fátima Â. (2011). Schiff Bases: A Short Review of Their Antimicrobial Activities. J. Adv. Res..

[51] Gavalyan V.B. (2016). Synthesis and Characterization of New Chitosan-Based Schiff Base Compounds. Carbohydr. Polym..

[52] Yin N., Santos T.M.A., Auer G.K., Crooks J.A., Oliver P.M., Weibel D.B. (2014). Bacterial Cellulose as a Substrate for Microbial Cell Culture. Appl. Environ. Microbiol..

[53] Oryan A., Kamali A., Moshiri A., Baharvand H., Daemi H. (2018). Chemical Crosslinking of Biopolymeric Scaffolds: Current Knowledge and Future Directions of Crosslinked Engineered Bone Scaffolds. Int. J. Biol. Macromol..

[54] Sacco P., Furlani F., De Marzo G., Marsich E., Paoletti S., Donati I. (2018). Concepts for Developing Physical Gels of Chitosan and of Chitosan Derivatives. Gels.

[55] Xue X., Hu Y., Wang S., Chen X., Jiang Y., Su J. (2022). Fabrication of Physical and Chemical Crosslinked Hydrogels for Bone Tissue Engineering. Bioact. Mater..

[56] Kirschning A., Dibbert N., Dräger G. (2018). Chemical Functionalization of Polysaccharides—Towards Biocompatible Hydrogels for Biomedical Applications. Chemistry.

[57] Zarzycki R., Modrzejewska Z., Nawrotek K. (2010). Drug Release from Hydrogel Matrices. Ecol. Chem. Eng..

[58] Shahinpoor M. (1992). Conceptual Design, Kinematics and Dynamics of Swimming Robotic Structures Using Ionic Polymeric Gel Muscles. Smart Mater. Struct..

[59] Bordbar-Khiabani A., Gasik M. (2022). Smart Hydrogels for Advanced Drug Delivery Systems. Int. J. Mol. Sci..

[60] AliakbarAhovan Z., Esmaeili Z., Eftekhari B.S., Khosravimelal S., Alehosseini M., Orive G., Dolatshahi-Pirouz A., Pal Singh Chauhan N., Janmey P.A., Hashemi A. (2022). Antibacterial Smart Hydrogels: New Hope for Infectious Wound Management. Mater. Today Bio..

[61] Rani Raju N., Silina E., Stupin V., Manturova N., Chidambaram S.B., Achar R.R. (2022). Multifunctional and Smart Wound Dressings—A Review on Recent Research Advancements in Skin Regenerative Medicine. Pharmaceutics.

[62] Mantha S., Pillai S., Khayambashi P., Upadhyay A., Zhang Y., Tao O., Pham H.M., Tran S.D. (2019). Smart Hydrogels in Tissue Engineering and Regenerative Medicine. Materials.

[63] Chopra H., Kumar S., Singh I. (2020). Bioadhesive Hydrogels and Their Applications. Bioadhesives in Drug Delivery.

[64] Xiong Y., Zhang X., Ma X., Wang W., Yan F., Zhao X., Chu X., Xu W., Sun C. (2021). A Review of the Properties and Applications of Bioadhesive Hydrogels. Polym. Chem..

[65] Sundriyal P., Pandey M., Bhattacharya S. (2020). Plasma-Assisted Surface Alteration of Industrial Polymers for Improved Adhesive Bonding. Int. J. Adhes. Adhes..

[66] Zheng K., Tong Y., Zhang S., He R., Xiao L., Iqbal Z., Zhang Y., Gao J., Zhang L., Jiang L. (2021). Flexible Bicolorimetric Polyacrylamide/Chitosan Hydrogels for Smart Real-Time Monitoring and Promotion of Wound Healing. Adv. Funct. Mater..

[67] Jahanmir G., Chau Y., Grumezescu A.M. (2019). Chapter 9–Mathematical Models of Drug Release from Degradable Hydrogels. Biomedical Applications of Nanoparticles.

[68] Vasile C., Pamfil D., Stoleru E., Baican M. (2020). New Developments in Medical Applications of Hybrid Hydrogels Containing Natural Polymers. Molecules.

[69] Fan W., Yin J., Yi C., Xia Y., Nie Z., Sui K. (2020). Nature-Inspired Sequential Shape Transformation of Energy-Patterned Hydrogel Sheets. ACS Appl. Mater. Interfaces.

[70] Li Y., Wang X., Fu Y., Wei Y., Zhao L., Tao L. (2018). Self-Adapting Hydrogel to Improve the Therapeutic Effect in Wound-Healing. ACS Appl. Mater. Interfaces.

[71] Wang Y., Xie R., Li Q., Dai F., Lan G., Shang S., Lu F. (2020). A Self-Adapting Hydrogel Based on Chitosan/Oxidized KonjacGlucomannan/AgNPs for Repairing Irregular Wounds. Biomater. Sci..

[72] Le T.M.D., Duong H.T.T., Thambi T., Giang Phan V.H., Jeong J.H., Lee D.S. (2018). Bioinspired PH- and Temperature-Responsive Injectable Adhesive Hydrogels with Polyplexes Promotes Skin Wound Healing. Biomacromolecules.

[73] Pham L., Dang L.H., Truong M.D., Nguyen T.H., Le L., Le V.T., Nam N.D., Bach L.G., Nguyen V.T., Tran N.Q. (2019). A Dual Synergistic of Curcumin and Gelatin on Thermal-Responsive Hydrogel Based on Chitosan-P123 in Wound Healing Application. Biomed. Pharmacother..

[74] Wang J., Zhao B., Sun L., Jiang L., Li Q., Jin P. (2022). Smart Thermosensitive Poloxamer Hydrogels Loaded with Nr-CWs for the Treatment of Diabetic Wounds. PLoS ONE.

[75] Paneysar J.S., Barton S., Ambre P., Coutinho E. (2022). Novel Temperature Responsive Films Impregnated with Silver Nano Particles (Ag-NPs) as Potential Dressings for Wounds. J. Pharm. Sci..

[76] Zhou L., Pi W., Cheng S., Gu Z., Zhang K., Min T., Zhang W., Du H., Zhang P., Wen Y. (2021). Multifunctional DNA Hydrogels with Hydrocolloid-Cotton Structure for Regeneration of Diabetic Infectious Wounds. Adv. Funct. Mater..

[77] Si Y., Wang L., Wang X., Tang N., Yu J., Ding B. (2017). Ultrahigh-Water-Content, Superelastic, and Shape-Memory Nanofiber-Assembled Hydrogels Exhibiting Pressure-Responsive Conductivity. Adv. Mater..

[78] Li D., Fei X., Xu L., Wang Y., Tian J., Li Y. (2022). Pressure-Sensitive Antibacterial Hydrogel Dressing for Wound Monitoring in Bed Ridden Patients. J. Colloid Interface Sci..

[79] de Alencar Fonseca Santos J., Campelo M.B.D., de Oliveira R.A., Nicolau R.A., Rezende V.E.A., Arisawa E.Â.L. (2018). Effects of Low-Power Light Therapy on the Tissue Repair Process of Chronic Wounds in Diabetic Feet. Photomed. Laser Surg..

[80] Lipovsky A., Nitzan Y., Lubart R. (2008). A Possible Mechanism for Visible Light-Induced Wound Healing. Lasers Surg. Med..

[81] Maiz-Fernández S., Pérez-Álvarez L., Ruiz-Rubio L., Vilas-Vilela J.L., Lanceros-Méndez S., Costa P., Costa C.M., Lanceros-Mendez S. (2021). Chapter Twelve–Multifunctional Materials Based on Smart Hydrogels for Biomedical and 4D Applications. Advanced Lightweight Multifunctional Materials.

[82] Yang N., Zhu M., Xu G., Liu N., Yu C. (2020). A Near-Infrared Light-Responsive Multifunctional Nanocomposite Hydrogel for Efficient and Synergistic Antibacterial Wound Therapy and Healing Promotion. J. Mater. Chem. B.

[83] Gupta A., Kowalczuk M., Heaselgrave W., Britland S.T., Martin C., Radecka I. (2019). The Production and Application of Hydrogels for Wound Management: A Review. Eur. Polym. J..

[84] Arenbergerova M., Arenberger P., Bednar M., Kubat P., Mosinger J. (2012). Light-Activated Nanofibre Textiles Exert Antibacterial Effects in the Setting of Chronic Wound Healing. Exp. Dermatol..

[85] Mai B., Jia M., Liu S., Sheng Z., Li M., Gao Y., Wang X., Liu Q., Wang P. (2020). Smart Hydrogel-Based DVDMS/BFGF Nanohybrids for Antibacterial Phototherapy with Multiple Damaging Sites and Accelerated Wound Healing. ACS Appl. Mater. Interfaces.

[86] Lv H., Liu J., Zhen C., Wang Y., Wei Y., Ren W., Shang P. (2021). Magnetic Fields as a Potential Therapy for Diabetic Wounds Based on Animal Experiments and Clinical Trials. Cell Prolif..

[87] Ekici Y., Aydogan C., Balcik C., Haberal N., Kirnap M., Moray G., Haberal M. (2012). Effect of Static Magnetic Field on Experimental Dermal Wound Strength. Indian J. Plast. Surg..

[88] Nezami S., Sadeghi M., Mohajerani H. (2020). A Novel PH-Sensitive and Magnetic Starch-Based Nanocomposite Hydrogel as a Controlled Drug Delivery System for Wound Healing. Polym. Degrad. Stab..

[89] Yang X., Zhang C., Deng D., Gu Y., Wang H., Zhong Q. (2022). Multiple Stimuli-Responsive MXene-Based Hydrogel as Intelligent Drug Delivery Carriers for Deep Chronic Wound Healing. Small.

[90] Xu J., Tsai Y.-L., Hsu S. (2020). Design Strategies of Conductive Hydrogel for Biomedical Applications. Molecules.

[91] Verdes M., Mace K., Margetts L., Cartmell S. (2022). Status and Challenges of Electrical Stimulation Use in Chronic Wound Healing. Curr. Opin. Biotechnol..

[92] Tai G., Tai M., Zhao M. (2018). Electrically Stimulated Cell Migration and Its Contribution to Wound Healing. Burns Trauma..

[93] Deng Z., Yu R., Guo B. (2021). Stimuli-Responsive Conductive Hydrogels: Design, Properties, and Applications. Mater. Chem. Front..

[94] Liang Y., Zhao X., Hu T., Chen B., Yin Z., Ma P.X., Guo B. (2019). Adhesive Hemostatic Conducting Injectable Composite Hydrogels with Sustained Drug Release and Photothermal Antibacterial Activity to Promote Full-Thickness Skin Regeneration during Wound Healing. Small Weinh. Bergstr. Ger..

[95] Zhang S., Ge G., Qin Y., Li W., Dong J., Mei J., Ma R., Zhang X., Bai J., Zhu C. (2023). Recent Advances in Responsive Hydrogels for Diabetic Wound Healing. Mater. Today Bio.

[96] Wang L., Zhou M., Xu T., Zhang X. (2022). Multifunctional Hydrogel as Wound Dressing for Intelligent Wound Monitoring. Chem. Eng. J..

[97] Ninan N., Forget A., Shastri V.P., Voelcker N.H., Blencowe A. (2016). Antibacterial and Anti-Inflammatory PH-Responsive Tannic Acid-Carboxylated Agarose Composite Hydrogels for Wound Healing. ACS Appl. Mater. Interfaces.

[98] Shi W., Kong Y., Su Y., Kuss M.A., Jiang X., Li X., Xie J., Duan B. (2021). Tannic Acid-Inspired, Self-Healing, and Dual Stimuli Responsive Dynamic Hydrogel with Potent Antibacterial and Anti-Oxidative Properties. J. Mater. Chem. B.

[99] Zhou Z., Zhang X., Xu L., Lu H., Chen Y., Wu C., Hu P. (2022). A Self-Healing Hydrogel Based on Crosslinked Hyaluronic Acid and Chitosan to Facilitate Diabetic Wound Healing. Int. J. Biol. Macromol..

[100] Li L., Lei D., Zhang J., Xu L., Li J., Jin L., Pan L. (2022). Dual-Responsive Alginate Hydrogel Constructed by Sulfhdryl Dendrimer as an Intelligent System for Drug Delivery. Molecules.

[101] Zhang J., Hurren C., Lu Z., Wang D. (2022). PH-Sensitive Alginate Hydrogel for Synergistic Anti-Infection. Int. J. Biol. Macromol..

[102] Qiao B., Pang Q., Yuan P., Luo Y., Ma L. (2020). Smart Wound Dressing for Infection Monitoring and NIR-Triggered Antibacterial Treatment. Biomater. Sci..

[103] Guo P., Liang J., Li Y., Lu X., Fu H., Jing H., Guan S., Han D., Niu L. (2019). High-Strength and PH-Responsive Self-Healing Polyvinyl Alcohol/Poly 6-Acrylamidohexanoic Acid Hydrogel Based on Dual Physically Cross-Linked Network. Colloids Surf. A Physicochem. Eng. Asp..

[104] Lanzalaco S., Mingot J., Torras J., Alemán C., Armelin E. (2023). Recent Advances in Poly(N-isopropylacrylamide) Hydrogels and Derivatives as Promising Materials for Biomedical and Engineering Emerging Applications. Adv. Eng. Mater..

[105] Han X., Yang R., Wan X., Dou J., Yuan J., Chi B., Shen J. (2021). Antioxidant and Multi-Sensitive PNIPAAm/Keratin Double Network Gels for Self-Stripping Wound Dressing Application. J. Mater. Chem. B.

[106] Qi X., Tong X., You S., Mao R., Cai E., Pan W., Zhang C., Hu R., Shen J. (2022). Mild Hyperthermia-Assisted ROS Scavenging Hydrogels Achieve Diabetic Wound Healing. ACS Macro Lett..

[107] Gao F., Xiong Z. (2021). Reactive Oxygen Species Responsive Polymers for Drug Delivery Systems. Front. Chem..

[108] Yu J., Zhang R., Chen B., Liu X., Jia Q., Wang X., Yang Z., Ning P., Wang Z., Yang Y. (2022). Injectable Reactive Oxygen Species-Responsive Hydrogel Dressing with Sustained Nitric Oxide Release for Bacterial Ablation and Wound Healing. Adv. Funct. Mater..

[109] Hu C., Zhang F., Long L., Kong Q., Luo R., Wang Y. (2020). Dual-Responsive Injectable Hydrogels Encapsulating Drug-Loaded Micelles for on-Demand Antimicrobial Activity and Accelerated Wound Healing. J. Control. Release.

[110] Qiu X., Zhang J., Cao L., Jiao Q., Zhou J., Yang L., Zhang H., Wei Y. (2021). Antifouling Antioxidant Zwitterionic Dextran Hydrogels as Wound Dressing Materials with Excellent Healing Activities. ACS Appl. Mater. Interfaces..

[111] Bas Y., Sanyal R., Sanyal A. (2023). Hyaluronic-Acid Based Redox-Responsive Hydrogels Using the Diels-Alder Reaction for on-Demand Release of Biomacromolecules. J. Macromol. Sci. Part A.

[112] Webber M.J., Anderson D.G. (2015). Smart Approaches to Glucose-Responsive Drug Delivery. J. Drug Target.

[113] Xu Z., Liu G., Huang J., Wu J. (2022). Novel Glucose-Responsive Antioxidant Hybrid Hydrogel for Enhanced Diabetic Wound Repair. ACS Appl. Mater. Interfaces.

[114] Zhou W., Duan Z., Zhao J., Fu R., Zhu C., Fan D. (2022). Glucose and MMP-9 Dual-Responsive Hydrogel with Temperature Sensitive Self-Adaptive Shape and Controlled Drug Release Accelerates Diabetic Wound Healing. Bioact. Mater..

[115] Yang J., Zeng W., Xu P., Fu X., Yu X., Chen L., Leng F., Yu C., Yang Z. (2022). Glucose-Responsive Multifunctional Metal–Organic Drug-Loaded Hydrogel for Diabetic Wound Healing. Acta Biomater..

[116] Gardikiotis I., Cojocaru F.-D., Mihai C.-T., Balan V., Dodi G. (2022). Borrowing the Features of Biopolymers for Emerging Wound Healing Dressings: A Review. Int. J. Mol. Sci..

[117] Naomi R., Bahari H., Ridzuan P.M., Othman F. (2021). Natural-Based Biomaterial for Skin Wound Healing (Gelatin vs. Collagen): Expert Review. Polymers.

[118] Chen J., He J., Yang Y., Qiao L., Hu J., Zhang J., Guo B. (2022). Antibacterial Adhesive Self-Healing Hydrogels to Promote Diabetic Wound Healing. Acta Biomater..

[119] Vahedi M., Barzin J., Shokrolahi F., Shokrollahi P. (2018). Self-Healing, Injectable Gelatin Hydrogels Cross-Linked by Dynamic Schiff Base Linkages Support Cell Adhesion and Sustained Release of Antibacterial Drugs. Macromol. Mater. Eng..

[120] Gutierrez-Reyes J.E., Caldera-Villalobos M., Claudio-Rizo J.A., Cabrera-Munguía D.A., Becerra-Rodriguez J.J., Soriano-Corral F., Herrera-Guerrero A. (2023). Smart Collagen/Xanthan Gum-Based Hydrogels with Antibacterial Effect, Drug Release Capacity and Excellent Performance in Vitro Bioactivity for Wound Healing Application. Biomed. Mater..

[121] Hu C., Liu W., Long L., Wang Z., Yuan Y., Zhang W., He S., Wang J., Yang L., Lu L. (2021). Microenvironment-Responsive Multifunctional Hydrogels with Spatiotemporal Sequential Release of Tailored Recombinant Human Collagen Type III for the Rapid Repair of Infected Chronic Diabetic Wounds. J. Mater. Chem. B.

[122] Yu X., Cheng C., Peng X., Zhang K., Yu X. (2022). A Self-Healing and Injectable Oxidized Quaternized Guar Gum/Carboxymethyl Chitosan Hydrogel with Efficient Hemostatic and Antibacterial Properties for Wound Dressing. Colloids Surf. B Biointerfaces.

[123] Almine J.F., Wise S.G., Weiss A.S. (2012). Elastin Signaling in Wound Repair. Birth Defects Res. Part C Embryo Today Rev..

[124] Kawabata S., Kanda N., Hirasawa Y., Noda K., Matsuura Y., Suzuki S., Kawai K. (2018). The Utility of Silk-Elastin Hydrogel as a New Material for Wound Healing. Plast. Reconstr. Surg.–Glob. Open.

[125] Stojic M., Ródenas-Rochina J., López-Donaire M.L., González De Torre I., González Pérez M., Rodríguez-Cabello J.C., Vojtová L., Jorcano J.L., Velasco D. (2021). Elastin-Plasma Hybrid Hydrogels for Skin Tissue Engineering. Polymers.

[126] Zhang Y., Desai M.S., Wang T., Lee S.-W. (2020). Elastin-Based Thermoresponsive Shape-Memory Hydrogels. Biomacromolecules.

[127] Zhang S., Shah S.A.-M., Basharat K., Qamar S.A., Raza A., Mohamed A., Bilal M., Iqbal H.M.N. (2022). Silk-Based Nano-Hydrogels for Futuristic Biomedical Applications. J. Drug Deliv. Sci. Technol..

[128] Mazurek Ł., Szudzik M., Rybka M., Konop M. (2022). Silk Fibroin Biomaterials and Their Beneficial Role in Skin Wound Healing. Biomolecules.

[129] Zheng H., Zuo B. (2021). Functional Silk Fibroin Hydrogels: Preparation, Properties and Applications. J. Mater. Chem. B.

[130] Römer L., Scheibel T. (2008). The Elaborate Structure of Spider Silk: Structure and Function of a Natural High Performance Fiber. Prion.

[131] Chang C., Zhang L. (2011). Cellulose-Based Hydrogels: Present Status and Application Prospects. Carbohydr. Polym..

[132] Lin F., Zheng J., Guo W., Zhu Z., Wang Z., Dong B., Lin C., Huang B., Lu B. (2019). Smart Cellulose-Derived Magnetic Hydrogel with Rapid Swelling and Deswelling Properties for Remotely Controlled Drug Release. Cellulose.

[133] SCOGS (Select Committee on GRAS Substances) https://www.cfsanappsexternal.fda.gov/scripts/fdcc/?set=SCOGS&sort=Sortsubstance&order=ASC&startrow=1&type=basic&search=ALGINATE.

[134] Inactive Ingredient Search for Approved Drug Products. https://www.accessdata.fda.gov/scripts/cder/iig/index.cfm?event=BasicSearch.page.

[135] Soeiro V.C., Melo K.R.T., Alves M.G.C.F., Medeiros M.J.C., Grilo M.L.P.M., Almeida-Lima J., Pontes D.L., Costa L.S., Rocha H.A.O. (2016). Dextran: Influence of Molecular Weight in Antioxidant Properties and Immunomodulatory Potential. Int. J. Mol. Sci..

[136] Luanda A., Badalamoole V. (2023). Past, Present and Future of Biomedical Applications of Dextran-Based Hydrogels: A Review. Int. J. Biol. Macromol..

[137] Sun G., Zhang X., Shen Y.-I., Sebastian R., Dickinson L.E., Fox-Talbot K., Reinblatt M., Steenbergen C., Harmon J.W., Gerecht S. (2011). Dextran Hydrogel Scaffolds Enhance Angiogenic Responses and Promote Complete Skin Regeneration during Burn Wound Healing. Proc. Natl. Acad. Sci. USA.

[138] Zhu Q., Jiang M., Liu Q., Yan S., Feng L., Lan Y., Shan G., Xue W., Guo R. (2018). Enhanced Healing Activity of Burn Wound Infection by a Dextran-HA Hydrogel Enriched with Sanguinarine. Biomater. Sci..

[139] Zhou L., Zhou L., Wei C., Guo R. (2022). A Bioactive Dextran-Based Hydrogel Promote the Healing of Infected Wounds via Antibacterial and Immunomodulatory. Carbohydr. Polym..

[140] Lin S.P., Kung H.N., Tsai Y.S., Tseng T.N., Hsu K.D., Cheng K.C. (2017). Novel Dextran Modified Bacterial Cellulose Hydrogel Accelerating Cutaneous Wound Healing. Cellulose.

[141] Zhao X., Li P., Guo B., Ma P.X. (2015). Antibacterial and Conductive Injectable Hydrogels Based on Quaternized Chitosan-Graft-Polyaniline/Oxidized Dextran for Tissue Engineering. Acta Biomater..

[142] Zhang M., Chen G., Lei M., Lei J., Li D., Zheng H. (2021). A PH-Sensitive Oxidized-Dextran Based Double Drug-Loaded Hydrogel with High Antibacterial Properties. Int. J. Biol. Macromol..

[143] Wu S., Yang Y., Wang S., Dong C., Zhang X., Zhang R., Yang L. (2022). Dextran and Peptide-Based PH-Sensitive Hydrogel Boosts Healing Process in Multidrug-Resistant Bacteria-Infected Wounds. Carbohydr. Polym..

[144] Guo C., Wu Y., Li W., Wang Y., Kong Q. (2022). Development of a Microenvironment-Responsive Hydrogel Promoting Chronically Infected Diabetic Wound Healing through Sequential Hemostatic, Antibacterial, and Angiogenic Activities. ACS Appl. Mater. Interfaces.

[145] Crini G. (2019). Historical Review on Chitin and Chitosan Biopolymers. Environ. Chem. Lett..

[146] Kou S.G., Peters L., Mucalo M. (2022). Chitosan: A Review of Molecular Structure, Bioactivities and Interactions with the Human Body and Micro-Organisms. Carbohydr. Polym..

[147] Li Y., Wang X., Wei Y., Tao L. (2017). Chitosan-Based Self-Healing Hydrogel for Bioapplications. Chin. Chem. Lett..

[148] Iacob A.-T., Drăgan M., Ghețu N., Pieptu D., Vasile C., Buron F., Routier S., Giusca S.E., Caruntu I.-D., Profire L. (2018). Preparation, Characterization and Wound Healing Effects of New Membranes Based on Chitosan, Hyaluronic Acid and Arginine Derivatives. Polymers.

[149] Azad A.K., Sermsintham N., Chandrkrachang S., Stevens W.F. (2004). Chitosan Membrane as a Wound-Healing Dressing: Characterization and Clinical Application. J. Biomed. Mater. Res. Part B Appl. Biomater..

[150] Ong S.-Y., Wu J., Moochhala S.M., Tan M.-H., Lu J. (2008). Development of a Chitosan-Based Wound Dressing with Improved Hemostatic and Antimicrobial Properties. Biomaterials.

[151] Radwan-Pragłowska J., Piątkowski M., Deineka V., Janus Ł., Korniienko V., Husak E., Holubnycha V., Liubchak I., Zhurba V., Sierakowska A. (2019). Chitosan-Based Bioactive Hemostatic Agents with Antibacterial Properties—Synthesis and Characterization. Molecules.

[152] Moeini A., Pedram P., Makvandi P., Malinconico M., Gomez d’Ayala G. (2020). Wound Healing and Antimicrobial Effect of Active Secondary Metabolites in Chitosan-Based Wound Dressings: A Review. Carbohydr. Polym..

[153] Guo X., Sun T., Zhong R., Ma L., You C., Tian M., Li H., Wang C. (2018). Effects of Chitosan Oligosaccharides on Human Blood Components. Front. Pharmacol..

[154] AbdEl-Hack M.E., El-Saadony M.T., Shafi M.E., Zabermawi N.M., Arif M., Batiha G.E., Khafaga A.F., Abd El-Hakim Y.M., Al-Sagheer A.A. (2020). Antimicrobial and Antioxidant Properties of Chitosan and Its Derivatives and Their Applications: A Review. Int. J. Biol. Macromol..

[155] Dai T., Tanaka M., Huang Y.-Y., Hamblin M.R. (2011). Chitosan Preparations for Wounds and Burns: Antimicrobial and Wound-Healing Effects. Expert Rev. Anti Infect. Ther..

[156] Cao R., Yu H., Long H., Zhang H., Hao C., Shi L., Du Y., Jiao S., Guo A., Ma L. (2022). Low Deacetylation Degree Chitosan Oligosaccharide Protects against IL-1β Induced Inflammation and Enhances Autophagy Activity in Human Chondrocytes. J. Biomater. Sci. Polym. Ed..

[157] Satitsri S., Muanprasat C. (2020). Chitin and Chitosan Derivatives as Biomaterial Resources for Biological and Biomedical Applications. Molecules.

[158] Deng L., Wang B., Li W., Han Z., Chen S., Wang H. (2022). Bacterial Cellulose Reinforced Chitosan-Based Hydrogel with Highly Efficient Self-Healing and Enhanced Antibacterial Activity for Wound Healing. Int. J. Biol. Macromol..

[159] Li H., Wei X., Yi X., Tang S., He J., Huang Y., Cheng F. (2021). Antibacterial, Hemostasis, Adhesive, Self-Healing Polysaccharides-Based Composite Hydrogel Wound Dressing for the Prevention and Treatment of Postoperative Adhesion. Mater. Sci. Eng. C.

[160] Huang Y., Mu L., Zhao X., Han Y., Guo B. (2022). Bacterial Growth-Induced Tobramycin Smart Release Self-Healing Hydrogel for Pseudomonas Aeruginosa -Infected Burn Wound Healing. ACS Nano.

[161] Tatarusanu S.-M., Sava A., Profire B.-S., Pinteala T., Jitareanu A., Iacob A.-T., Lupascu F., Simionescu N., Rosca I., Profire L. (2023). New Smart Bioactive and Biomimetic Chitosan-Based Hydrogels for Wounds Care Management. Pharmaceutics.

[162] Castillo-Henríquez L., Castro-Alpízar J., Lopretti-Correa M., Vega-Baudrit J. (2021). Exploration of Bioengineered Scaffolds Composed of Thermo-Responsive Polymers for Drug Delivery in Wound Healing. Int. J. Mol. Sci..

[163] Rusu A.G., Chiriac A.P., Nita L.E., Rosca I., Pinteala M., Mititelu-Tartau L. (2020). Chitosan Derivatives in Macromolecular Co-Assembly Nanogels with Potential for Biomedical Applications. Biomacromolecules.

[164] Lee K.Y., Mooney D.J. (2012). Alginate: Properties and Biomedical Applications. Prog. Polym. Sci..

[165] Roquero D.M., Katz E. (2022). “Smart” Alginate Hydrogels in Biosensing, Bioactuation and Biocomputing: State-of-the-Art and Perspectives. Sens. Actuators Rep..

[166] Ueno M., Oda T. (2014). Biological Activities of Alginate. Adv. Food Nutr. Res..

[167] Xing M., Cao Q., Wang Y., Xiao H., Zhao J., Zhang Q., Ji A., Song S. (2020). Advances in Research on the Bioactivity of Alginate Oligosaccharides. Mar. Drugs..

[168] Kim N.-G., Kim S.-C., Kim T.-H., Je J.-Y., Lee B., Lee S.G., Kim Y.-M., Kang H.W., Qian Z.-J., Kim N. (2023). Ishophloroglucin A-Based Multifunctional Oxidized Alginate/Gelatin Hydrogel for Accelerating Wound Healing. Int. J. Biol. Macromol..

[169] Liu L., Li X., Nagao M., Elias A., Narain R., Chung H.-J. (2017). A PH-Indicating Colorimetric Tough Hydrogel Patch towards Applications in a Substrate for Smart Wound Dressings. Polymers.

[170] Chou H.-Y., Weng C.-C., Lai J.-Y., Lin S.-Y., Tsai H.-C. (2020). Design of an Interpenetrating Polymeric Network Hydrogel Made of Calcium-Alginate from a Thermos-Sensitive Pluronic Template as a Thermal-Ionic Reversible Wound Dressing. Polymers.

[171] Yao Z., Qian Y., Jin Y., Wang S., Li J., Yuan W.-E., Fan C. (2022). Biomimetic Multilayer Polycaprolactone/Sodium Alginate Hydrogel Scaffolds Loaded with Melatonin Facilitate Tendon Regeneration. Carbohydr. Polym..

[172] Xu K., Deng S., Zhu Y., Yang W., Chen W., Huang L., Zhang C., Li M., Ao L., Jiang Y. (2023). Platelet Rich Plasma Loaded Multifunctional Hydrogel Accelerates Diabetic Wound Healing via Regulating the Continuously Abnormal Microenvironments. Adv. Healthc. Mater..

[173] Fallacara A., Baldini E., Manfredini S., Vertuani S. (2018). Hyaluronic Acid in the Third Millennium. Polymers..

[174] Ke C., Sun L., Qiao D., Wang D., Zeng X. (2011). Antioxidant Acitivity of Low Molecular Weight Hyaluronic Acid. Food Chem. Toxicol. Int. J. Publ. Br. Ind. Biol. Res. Assoc..

[175] Litwiniuk M., Krejner A., Speyrer M.S., Gauto A.R., Grzela T. (2016). Hyaluronic Acid in Inflammation and Tissue Regeneration. Wounds Compend. Clin. Res. Pract..

[176] Yang H., Song L., Zou Y., Sun D., Wang L., Yu Z., Guo J. (2021). Role of Hyaluronic Acids and Potential as Regenerative Biomaterials in Wound Healing. ACS Appl. Bio Mater..

[177] de Paiva W.K.V., de Medeiros W.R.D.B., de Assis C.F., Dos Santos E.S., de Sousa Júnior F.C. (2022). Physicochemical Characterization and in Vitro Antioxidant Activity of Hyaluronic Acid Produced by *Streptococcus zooepidemicus* CCT 7546. Prep. Biochem. Biotechnol..

[178] Hussain Z., Thu H.E., Katas H., Bukhari S.N.A. (2017). Hyaluronic Acid-Based Biomaterials: A Versatile and Smart Approach to Tissue Regeneration and Treating Traumatic, Surgical, and Chronic Wounds. Polym. Rev..

[179] Ebid R., Lichtnekert J., Anders H.-J. (2014). Hyaluronan Is Not a Ligand but a Regulator of Toll-Like Receptor Signaling in Mesangial Cells: Role of Extracellular Matrix in Innate Immunity. ISRN Nephrol..

[180] Ding Y.-W., Wang Z.-Y., Ren Z.-W., Zhang X.-W., Wei D.-X. (2022). Advances in Modified Hyaluronic Acid-Based Hydrogels for Skin Wound Healing. Biomater. Sci..

[181] Makvandi P., Caccavale C., Della Sala F., Zeppetelli S., Veneziano R., Borzacchiello A. (2020). Natural Formulations Provide Antioxidant Complement to Hyaluronic Acid-Based Topical Applications Used in Wound Healing. Polymers.

[182] Nyman E., Henricson J., Ghafouri B., Anderson C.D., Kratz G. (2019). Hyaluronic Acid Accelerates Re-Epithelialization and Alters Protein Expression in a Human Wound Model. Plast. Reconstr. Surg. Glob. Open.

[183] Guan S., Li Y., Cheng C., Gao X., Gu X., Han X., Ye H. (2020). Manufacture of PH- and HAase-Responsive Hydrogels with on-Demand and Continuous Antibacterial Activity for Full-Thickness Wound Healing. Int. J. Biol. Macromol..

[184] Deng M., Wu Y., Ren Y., Song H., Zheng L., Lin G., Wen X., Tao Y., Kong Q., Wang Y. (2022). Clickable and Smart Drug Delivery Vehicles Accelerate the Healing of Infected Diabetic Wounds. J. Control. Release.

[185] Das S., Das D. (2021). Rational Design of Peptide-Based Smart Hydrogels for Therapeutic Applications. Front. Chem..

[186] Guan T., Li J., Chen C., Liu Y. (2022). Self-Assembling Peptide-Based Hydrogels for Wound Tissue Repair. Adv. Sci..

[187] Chen J., Zou X. (2019). Self-Assemble Peptide Biomaterials and Their Biomedical Applications. Bioact. Mater..

[188] Ye Z., Zhang H., Luo H., Wang S., Zhou Q., Du X., Tang C., Chen L., Liu J., Shi Y.-K. (2008). Temperature and PH Effects on Biophysical and Morphological Properties of Self-Assembling Peptide RADA16-I. J. Pept. Sci..

[189] Ng V.W.L., Chan J.M.W., Sardon H., Ono R.J., García J.M., Yang Y.Y., Hedrick J.L. (2014). Antimicrobial Hydrogels: A New Weapon in the Arsenal against Multidrug-Resistant Infections. Adv. Drug Deliv. Rev..

[190] Schnaider L., Brahmachari S., Schmidt N.W., Mensa B., Shaham-Niv S., Bychenko D., Adler-Abramovich L., Shimon L.J.W., Kolusheva S., DeGrado W.F. (2017). Self-Assembling Dipeptide Antibacterial Nanostructures with Membrane Disrupting Activity. Nat. Commun..

[191] Ortega-Velázquez R., Díez-Marqués M.L., Ruiz-Torres M.P., González-Rubio M., Rodríguez-Puyol M., Rodríguez Puyol D. (2003). Arg-Gly-Asp-Ser (RGDS) Peptide Stimulates Transforming Growth Factor Beta1 Transcription and Secretion through Integrin Activation. FASEB J. Off. Publ. Fed. Am. Soc. Exp. Biol..

[192] Lee S., Trinh T.H.T., Yoo M., Shin J., Lee H., Kim J., Hwang E., Lim Y.B., Ryou C. (2019). Ryou Self-Assembling Peptides and Their Application in the Treatment of Diseases. Int. J. Mol. Sci..

[193] Sankar S., O’Neill K., Bagot D’Arc M., Rebeca F., Buffier M., Aleksi E., Fan M., Matsuda N., Gil E.S., Spirio L. (2021). Clinical Use of the Self-Assembling Peptide RADA16: A Review of Current and Future Trends in Biomedicine. Front. Bioeng. Biotechnol..

[194] PuraStat. https://3dmatrix.com/products/purastat/.

[195] Tian T., Li Y., Lin Y. (2022). Prospects and Challenges of Dynamic DNA Nanostructures in Biomedical Applications. Bone Res..

[196] Fu L., Li P., Zhu J., Liao Z., Gao C., Li H., Yang Z., Zhao T., Chen W., Peng Y. (2022). Tetrahedral Framework Nucleic Acids Promote the Biological Functions and Related Mechanism of Synovium-Derived Mesenchymal Stem Cells and Show Improved Articular Cartilage Regeneration Activity in Situ. Bioact. Mater..

[197] Jiang X., Li M., Guo X., Yang M., Rasooly A. (2019). Self-Assembled DNA-THPS Hydrogel as a Topical Antibacterial Agent for Wound Healing. ACS Appl. Bio Mater..

[198] Hutanu D. (2014). Recent Applications of Polyethylene Glycols (PEGs) and PEG Derivatives. Mod. Chem. Appl..

[199] Handbook of Pharmaceutical Excipients 6th Edition|PDF|Magnesium|Tablet (Pharmacy). https://www.scribd.com/document/246196761/Handbook-of-Pharmaceutical-Excipients-6th-Edition.

[200] Chen S.-L., Fu R.-H., Liao S.-F., Liu S.-P., Lin S.-Z., Wang Y.-C. (2018). A PEG-Based Hydrogel for Effective Wound Care Management. Cell Transplant..

[201] Chen Y., Li J., Lu J., Ding M., Chen Y. (2022). Synthesis and Properties of Poly(Vinyl Alcohol) Hydrogels with High Strength and Toughness. Polym. Test..

[202] Bodratti A., Alexandridis P. (2018). Formulation of Poloxamers for Drug Delivery. J. Funct. Biomater..

[203] Russo E., Villa C. (2019). Poloxamer Hydrogels for Biomedical Applications. Pharmaceutics.

[204] Akiyama H., Tamaoki N. (2007). Synthesis and Photoinduced Phase Transitions of Poly (N-Isopropylacrylamide) Derivative Functionalized with Terminal Azobenzene Units. Macromolecules.

[205] Duan Q., Miura Y., Narumi A., Shen X., Sato S.-I., Satoh T., Kakuchi T. (2006). Synthesis and Thermoresponsive Property of End-Functionalized Poly (N-Isopropylacrylamide) with Pyrenyl Group. J. Polym. Sci. Part Polym. Chem..

[206] Yu B., Chan J.W., Hoyle C.E., Lowe A.B. (2009). Sequential Thiol-Ene/Thiol-Ene and Thiol-Ene/Thiol-Yne Reactions as a Route to Well-Defined Mono and Bis End-Functionalized Poly (N-Isopropylacrylamide). J. Polym. Sci. Part Polym. Chem..

[207] Ansari M.J., Rajendran R.R., Mohanto S., Agarwal U., Panda K., Dhotre K., Manne R., Deepak A., Zafar A., Yasir M. (2022). Poly(N-Isopropylacrylamide)-Based Hydrogels for Biomedical Applications: A Review of the State-of-the-Art. Gels.

